# Supplementary material for: KIF11 prevents retinal endothelial ferroptosis in familial exudative vitreoretinopathy by inhibiting phosphorylation-driven PRDX1 phase separation
Source: Nat Commun. 2026 Mar 24;17:4360. doi: 10.1038/s41467-026-71009-7 (PMC13172007; doi:10.1038/s41467-026-71009-7)

Supplementary Materials for

KIF11 prevents retinal endothelial ferroptosis in familial exudative vitreoretinopathy by inhibiting phosphorylation-driven PRDX1 phase separation

Mu Yang[1,2,†], Rulian Zhao[3,†], Li Peng[1,†], Liting Lv[1,†], Lanyao Yang[1], Yunqi He[1], Xu Ha[1], Huijuan Xu[1], Xiang Zhang[4], Peiquan Zhao[4], Shujin Li[1,2*], Zhenglin Yang[1,2,5*]

[1]Genetic Diseases Key Laboratory of Sichuan Province, Department of Medical Genetics, Sichuan Academy of Medical Sciences & Sichuan Provincial People's Hospital, School of Medicine, University of Electronic Science and Technology of China, 610072 Chengdu, PR China
[2]Sichuan-Chongqing Joint Key Laboratory of Pathology and Laboratory Medicine, Jinfeng Laboratory, 401329 Chongqing, PR China
[3]Department of Ophthalmology, Sichuan Academy of Medical Sciences & Sichuan Provincial People's Hospital, School of Medicine, University of Electronic Science and Technology of China, 610072 Chengdu, PR China
[4]Department of Ophthalmology, Xin Hua Hospital Affiliated to Shanghai Jiao Tong University School of Medicine, 200092 Shanghai, PR China
[5]Research Unit for Blindness Prevention, Chinese Academy of Medical Sciences (2019RU026), Sichuan Academy of Medical Sciences & Sichuan Provincial People's Hospital, 610072 Chengdu, PR China

These authors contributed equally: Mu Yang, Rulian Zhao, Li Peng, and Liting Lv.
These authors jointly supervised this work: Shujin Li, Zhenglin Yang.
e-mail: lishujin91@126.com (Shujin Li), yangzhenglin@cashq.ac.cn (Zhenglin Yang).

Contents

1. Supplementary Figure 1–14
2. Supplementary Table 1–6

Supplementary Figures

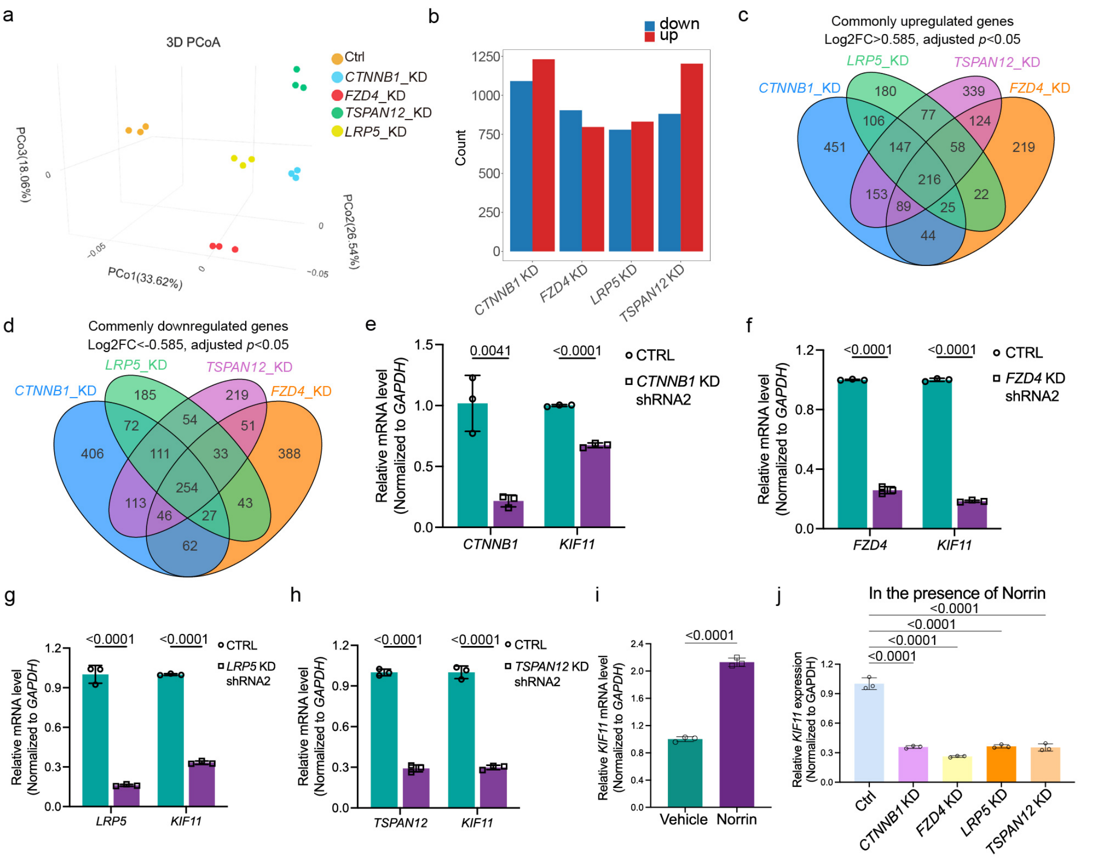

Supplementary Fig. 1 KIF11 is identified as a downstream target of Norrin/beta-catenin signaling pathway. a 3D PCoA summarizing the bulk RNA-seq distribution of control (CTRL), *CTNNB1* Knockdown (KD), *FZD4* KD, *LRP5* KD, and *TSPAN12* KD HRECs. **b** Differentially expressed genes (DEGs) in KD samples versus CTRL identified by bulk RNA-seq. **c**, **d** Venn analysis of commonly upregulated (**c**) and downregulated (**d**) DEGs in CTRL and KD HRECs ($|\log_2\text{FoldChange}| > 0.585$, adjusted $p < 0.05$). **e-h** Relative mRNA levels of *KIF11* and corresponding genes in CTRL, *CTNNB1*, *FZD4*, *LRP5*, or *TSPAN12* KD HRECs, each with an independent shRNA (shRNA2). $n = 3$. **i** Relative *KIF11* mRNA levels in the presence or absence of exogenous Norrin. $n = 3$. **j** Relative *KIF11* mRNA levels in HRECs with exogenous Norrin. $n = 3$. Data are presented as mean ± SD. n represents independent biological replicates. Statistical significance was determined using a two-tailed Student's t-test (**e-i**) or one-way ANOVA with Dunnett's multiple comparisons test (**j**). Source data are provided as a Source Data file.

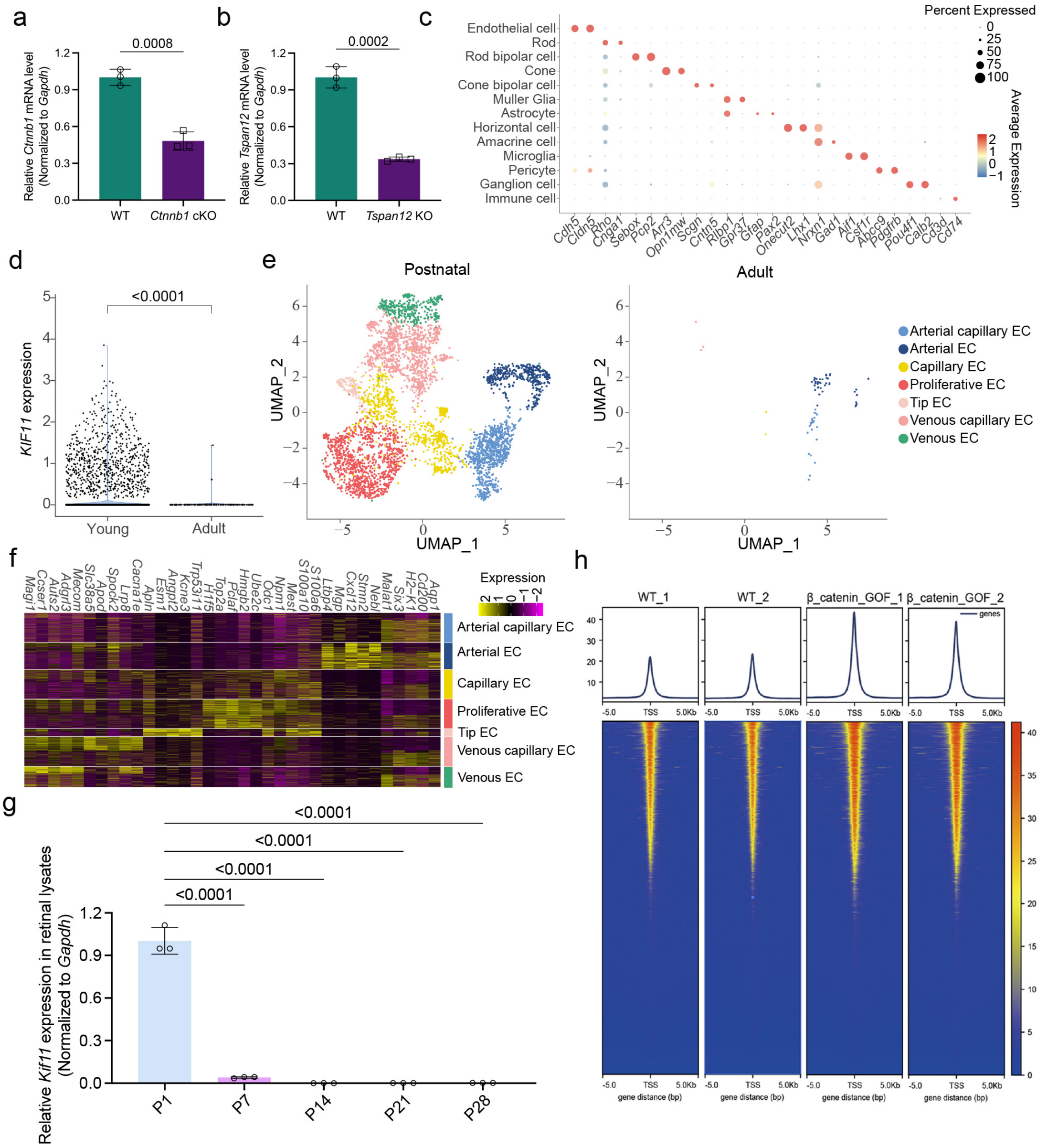

Supplementary Fig. 2 Predominant expression of *Kif11* in proliferative ECs. a, **b** Relative mRNA levels of *Ctnnb1* in P9 wild-type (WT) versus *Ctnnb1* cKO mice (**a**), and of P9 *Tspan12* in WT versus *Tspan12* KO mice (**b**) lung lysates. n = 3. **c** Dot plot of marker gene expression across annotated cell types. **d** Violin plots of *Kif11* expression in endothelial cells from postnatal (P6) and adult (P26 and P60) mice retinas. n = 5211 (Young) and 127 cells (Adult) per group. **e** UMAP of EC subclusters from postnatal and adult groups. **f** Heatmap of marker gene expression across EC subclusters. **g** Quantification of relative *Kif11* mRNA levels in retinas from P1, P7, P14, P21, and P28 mice. n = 3. **h** Heatmaps of ATAC-seq signal (chromatin accessibility) around the TSS in isolated cerebellar ECs from WT and EC-specific β-catenin-stabilized mice. Data are presented as mean ± SD. n represents independent biological replicates or the number of mice per group. Statistical significance was determined using a two-tailed

Student's t-test (**a**, **b**, **d**), or one-way ANOVA with Dunnett's multiple comparisons test (**g**). Source data are provided as a Source Data file.

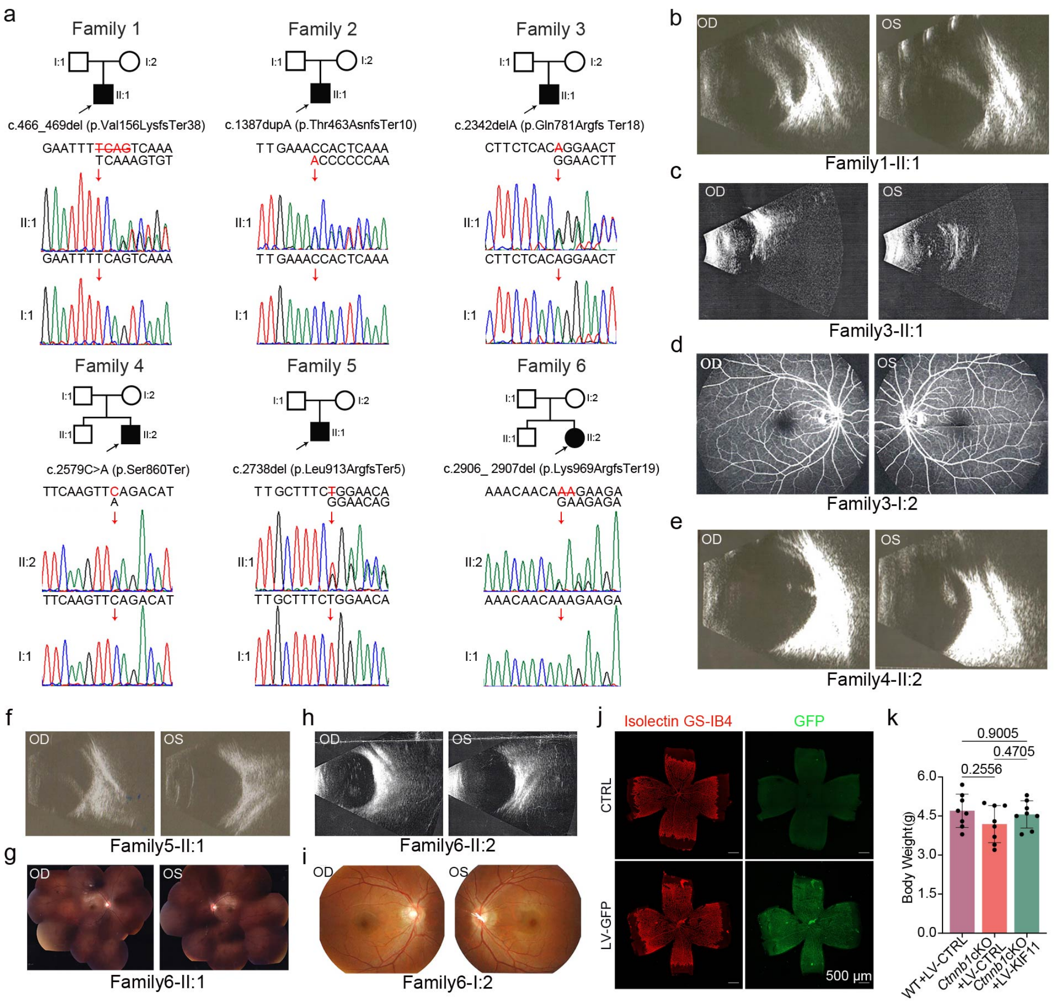

Supplementary Fig. 3 Identification of novel frameshift *KIF11* variants and evaluation of lentiviral delivery efficiency in mice. a Pedigrees and Sanger sequencing of FEVR families. The ages of the probands in Family 1–6 were 6 months, 3.8 years (46 months), 1.7 years (20 months), 8 months, 4.4 years (53 months), and 8 months, respectively. **b-i** Type-B ultrasonic imaging (**b**, **c**, **e**, **f**, **h**), fundus fluorescein angiography (**d**), and fundus photography (**g**, **i**) of probands and their unaffected relatives. **j** Representative images of retinal flat mounts from uninjected P7 mice and P7 mice retro-orbitally injected with non-targeting GFP-expressing lentivirus. Red, Isolectin GS-IB4; green, GFP. Scale bars, 500 μm. **k** Body weight of LV-CTRL-treated WT and LV-CTRL-, or LV-KIF11-treated *Ctnnb1* cKO mice. *n* = 8. Data are presented as mean ± SD. *n* represents the number of mice per group. Statistical significance was determined using one-way ANOVA with Tukey's (**k**) multiple comparisons test. Source data are provided as a Source Data file.

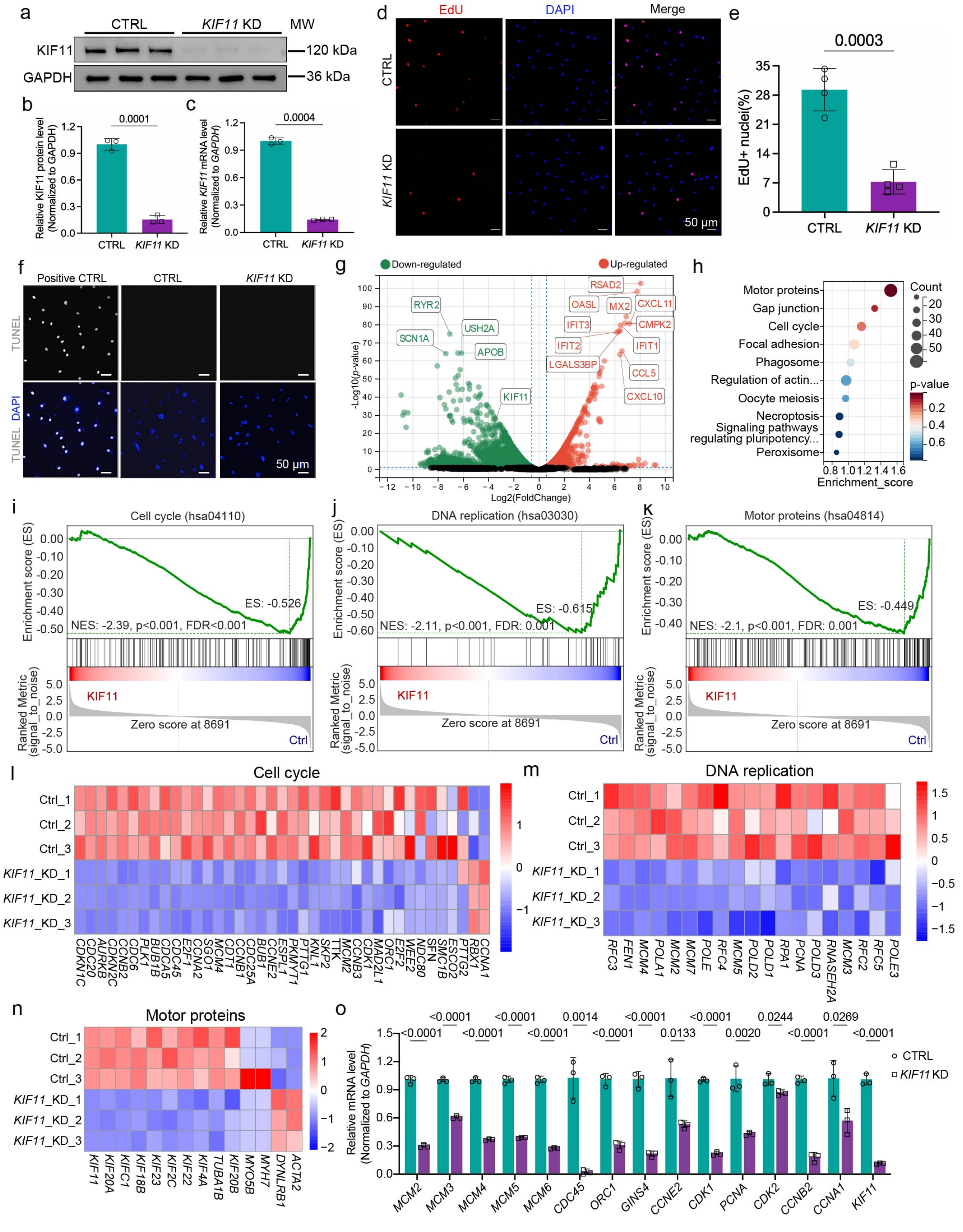

Supplementary Fig. 4 Loss of *KIF11* leads to negative regulation of pathways involved in cell proliferation. a-c Immunoblotting (**a**), relative protein (**b**), and mRNA levels (**c**) of KIF11 in CTRL and *KIF11* KD HRECs. *n* = 3. **d**, **e** Representative EdU staining images (**d**) and quantification of EdU+ cells (%) (**e**) in CTRL and *KIF11* KD HRECs. Red, EdU; blue, DAPI. Scale bars, 50 μm. *n* = 4. **f** Representative images of TUNEL+ cells in CTRL and *KIF11* KD HRECs. White, TUNEL; blue, DAPI. Scale bars, 50 μm. **g** Volcano plot of DEGs identified by bulk RNA-seq in CTRL and *KIF11* KD HRECs, with log_2FoldChange thresholds of -0.585 and 0.585 and a *p*-value

threshold of 0.05. **h** Bubble plot of KEGG pathway enrichment analysis of downregulated genes (log_2FoldChange < -1, $p < 0.05$) upon *KIF11* knockdown. **i-n** GSEA enrichment plots (**i-k**) and corresponding heatmaps (**l-n**) of KEGG cell cycle (**i**, **l**), DNA replication (**j**, **m**), and motor protein pathway (**k**, **n**) gene sets in CTRL versus *KIF11* KD HRECs. **o** Quantification of relative mRNA levels of genes involved in the cell cycle and DNA replication pathway. $n = 3$. Data are presented as mean ± SD. n represents independent biological replicates. Statistical significance was determined using two-tailed Student's t-test (**c**, **e**, **o**) or two-tailed Welch's t-test (**b**). Source data are provided as a Source Data file.

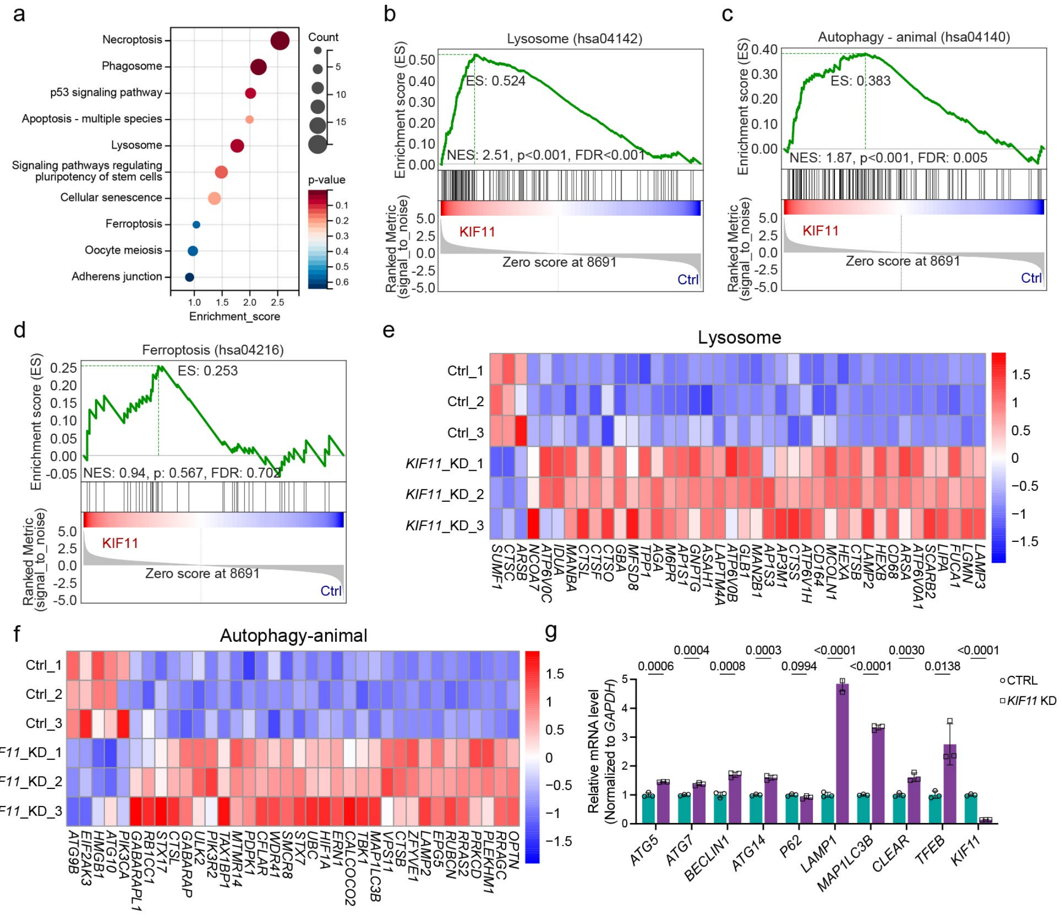

Supplementary Fig. 5 Bulk RNA-seq analysis reveals upregulation of pathways involved in phagosome and lysosome upon *KIF11* depletion. a Bubble plot of the KEGG pathway enrichment analysis of upregulated genes (|$\log_2$FoldChange| > 1, p < 0.05) upon *KIF11* knockdown. **b-d** GSEA enrichment plots of the KEGG lysosome (**b**), autophagy (**c**), and ferroptosis (**d**) signaling gene sets for the CTRL and *KIF11* KD HRECs. **e**, **f** Heatmaps of the KEGG lysosome (**e**) and autophagy (**f**) signaling gene sets for the CTRL and *KIF11* KD HRECs. **g** Relative quantification of mRNA levels of the autophagy- and lysosome-related gene. n = 3. Data are presented as mean ± SD. n represents independent biological replicates. Statistical significance was determined using two-tailed Student's t-test (**g**). Source data are provided as a Source Data file.

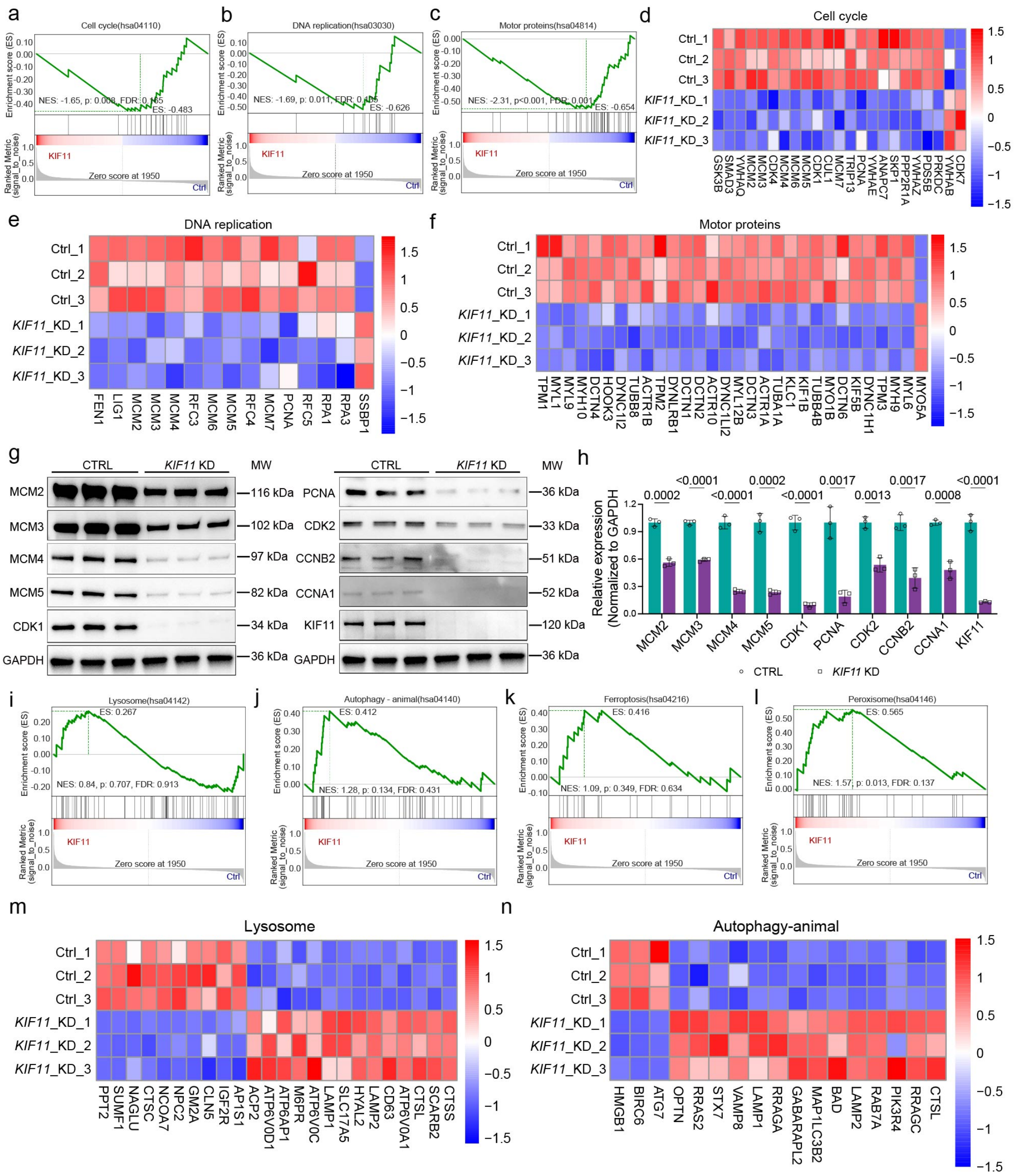

Supplementary Fig. 6 Proteomic analysis links *KIF11* depletion to reduced proliferation and enhanced autophagy/ferroptosis. a-c GSEA enrichment plots of the KEGG cell cycle (**a**), DNA replication (**b**), and motor protein (**c**) gene sets in CTRL versus *KIF11* KD HRECs. **d-f** Heatmaps of KEGG cell cycle (**d**), DNA replication (**e**), and motor protein (**f**) pathway gene sets in CTRL and *KIF11* KD HRECs. **g**, **h** Western blot analysis (**g**) and relative quantification of expression levels (**h**) of core genes involved in the cell cycle and DNA replication pathways in CTRL and *KIF11* KD HRECs. n = 3. **i-l** GSEA enrichment plots of the KEGG lysosome (**i**), autophagy (**j**), ferroptosis (**k**), and peroxisome (**l**) signaling gene sets in CTRL versus *KIF11* KD HRECs. **m**, **n** Heatmaps of KEGG lysosome (**m**) and autophagy (**n**) signaling gene sets in CTRL and *KIF11* KD HRECs. Data are presented as mean ± SD. n represents

independent biological replicates. Statistical significance was determined using two-tailed Student's t-test (**h**). Source data are provided as a Source Data file.

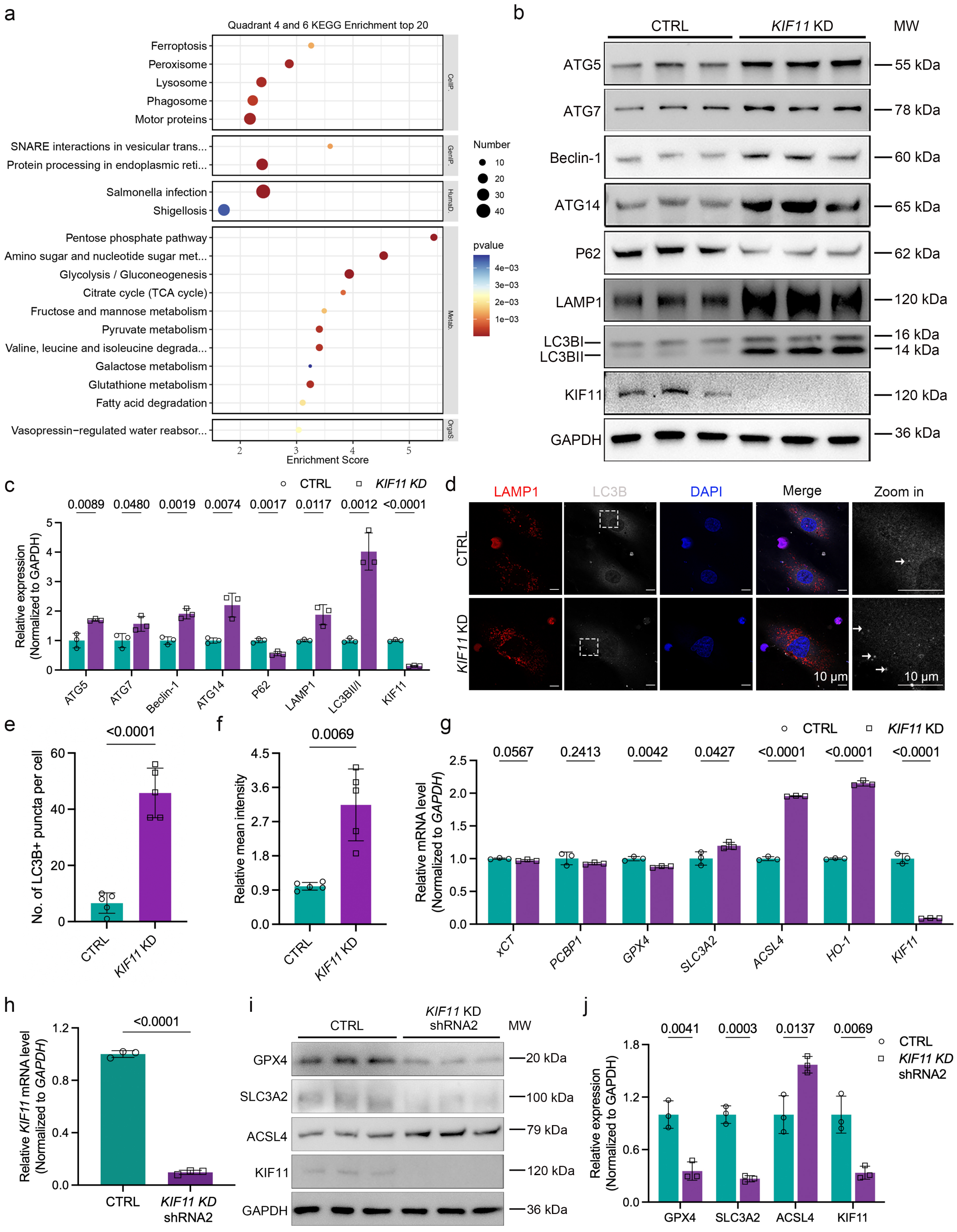

Supplementary Fig. 7 KIF11 deficiency induces cellular phenotypes of autophagy-accompanied ferroptosis. a Bubble plot of the KEGG pathway enrichment analysis for differentially expressed proteins with minimal changes at mRNA levels in *KIF11* KD cells. **b**, **c** Western blot analysis (**b**) and relative quantification (**c**) of autophagy- and lysosome-related proteins in CTRL and *KIF11* KD HRECs. *n* = 3. **d** Representative immunofluorescent images of LAMP1 and LC3B staining in CTRL and *KIF11* KD HRECs. Red, LAMP1; white, LC3B; blue, DAPI. Scale bars, 10 μm. **e** Relative quantification of the LC3B puncta numbers per cell in CTRL and *KIF11* KD HRECs.

n = 5. **f** Relative quantification of immunofluorescence intensity of LAMP1 in CTRL and *KIF11* KD HRECs. *n* = 5. **g** Relative mRNA levels of ferroptosis-related genes in CTRL and *KIF11* KD HRECs. *n* = 3. **h** Relative mRNA levels of *KIF11* in CTRL versus *KIF11* KD HRECs using an independent shRNA (shRNA2). *n* = 3. **i**, **j** Western blot analysis (**i**) and relative quantification (**j**) of KIF11 and core genes involved in ferroptosis in CTRL and *KIF11* KD HRECs using an independent shRNA. *n* = 3. Data are presented as mean ± SD. *n* represents independent biological replicates. Statistical significance was determined using two-tailed Student's t-test (**e**, **h**), two-tailed Welch's t-test (**f**), or one-way ANOVA with Tukey's multiple comparisons test (**c**, **g**, **j**). Source data are provided as a Source Data file.

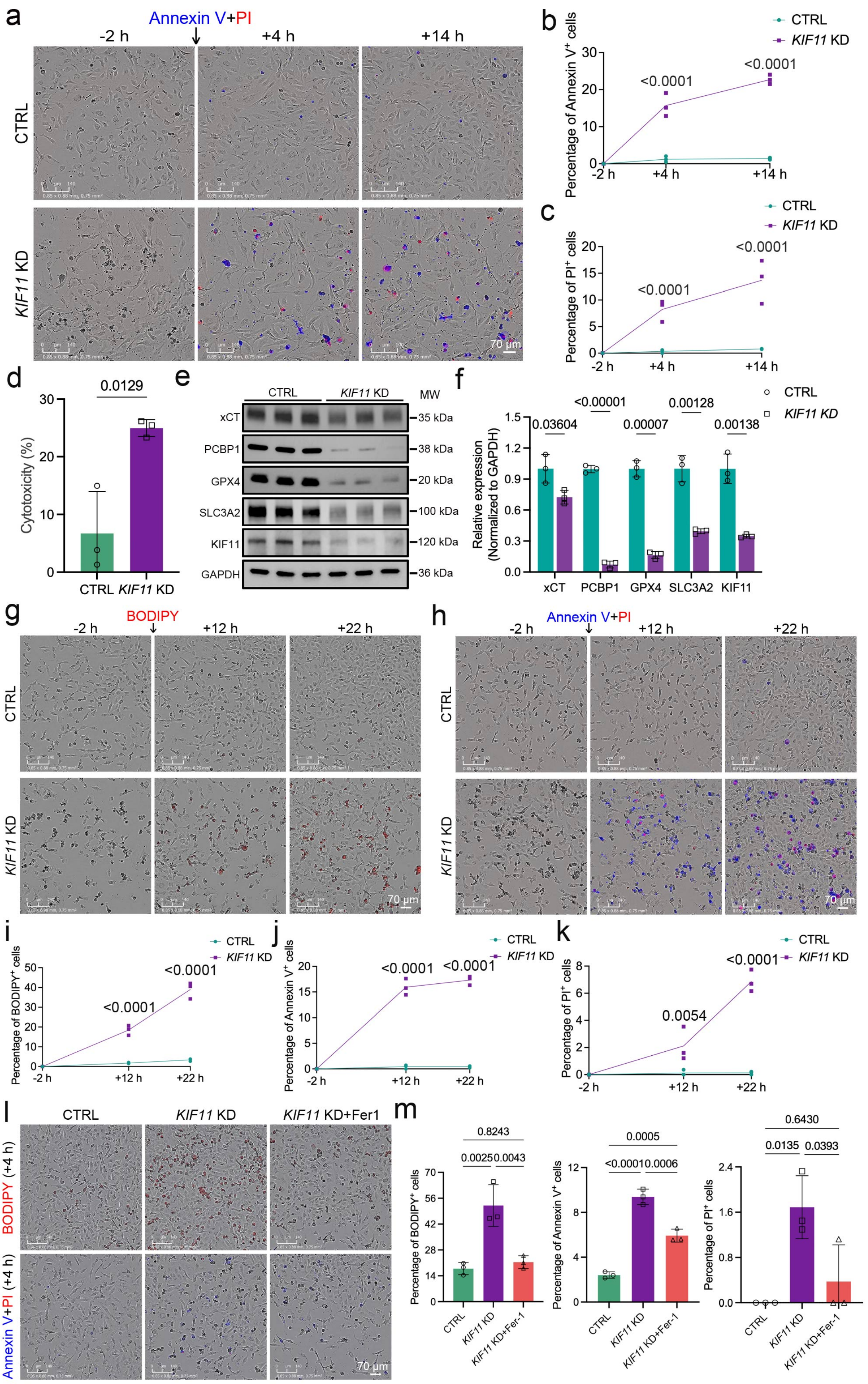

Supplementary Fig. 8 Loss of KIF11 induces ferroptosis in both HRECs and

HT1080 cells. a Representative time-lapse imaging of CTRL and *KIF11* KD HRECs stained with Annexin V (blue) and PI (red). Scale bars, 70 μm. **b**, **c** Quantification of Annexin V+ (**b**) and PI+ (**c**) cells from time-lapse imaging of CTRL and *KIF11* KD HRECs. *n* = 3. **d** Cytotoxicity (%) determined by LDH release assay in CTRL and *KIF11* KD HRECs. *n* = 3. **e**, **f** Western blot analysis (**e**) and relative quantification (**f**) of ferroptosis-related proteins in CTRL and *KIF11* KD HT1080 cells. *n* = 3. **g**, **h** Representative time-lapse images of CTRL and *KIF11* KD HT1080 cells stained with BODIPY (red, **g**), Annexin V (blue, **h**), and PI (red, **h**). Scale bars, 70 μm. **i-k** Quantification of BODIPY+ (**i**), Annexin V+ (**j**), and PI+ (**k**) cells from time-lapse imaging of CTRL and *KIF11* KD HT1080 cells. *n* = 3. **l**, **m** Representative images (**l**) and quantification (**m**) of BODIPY (red), Annexin V (blue), and PI (red) staining in CTRL and *KIF11* KD HT1080 cells treated with DMSO, and *KIF11* KD HT1080 cells treated with Fer-1 (10 μM). *n* = 3. Data are presented as mean ± SD. *n* represents independent biological replicates. Statistical significance was determined using two-tailed Student's t-test (**d**, **f**), one-way ANOVA with Tukey's multiple comparisons test (**m**), or two-way ANOVA with Sidak's multiple comparisons test (**b**, **c**, **i**, **j**, **k**). Source data are provided as a Source Data file.

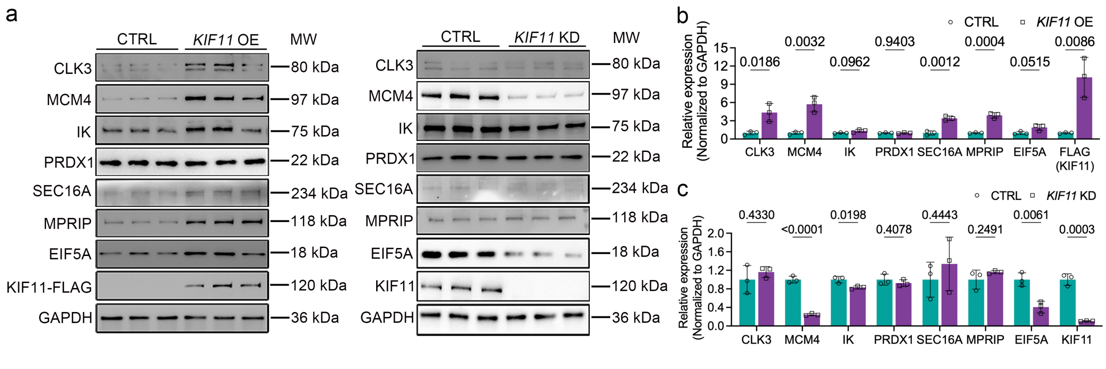

Supplementary Fig. 9 Protein levels of potential KIF11-binding proteins in KIF11 OE or KD HRECs. a Immunoblotting of potential binding partners of KIF11 upon *KIF11* overexpression or knockdown. **b**, **c** Quantification of relative expression levels upon *KIF11* overexpression (**b**) or depletion (**c**). $n = 3$. Data are presented as mean ± SD. n represents independent biological replicates. Statistical significance was determined using a two-tailed Student's t-test (**b**, **c**). Source data are provided as a Source Data file.

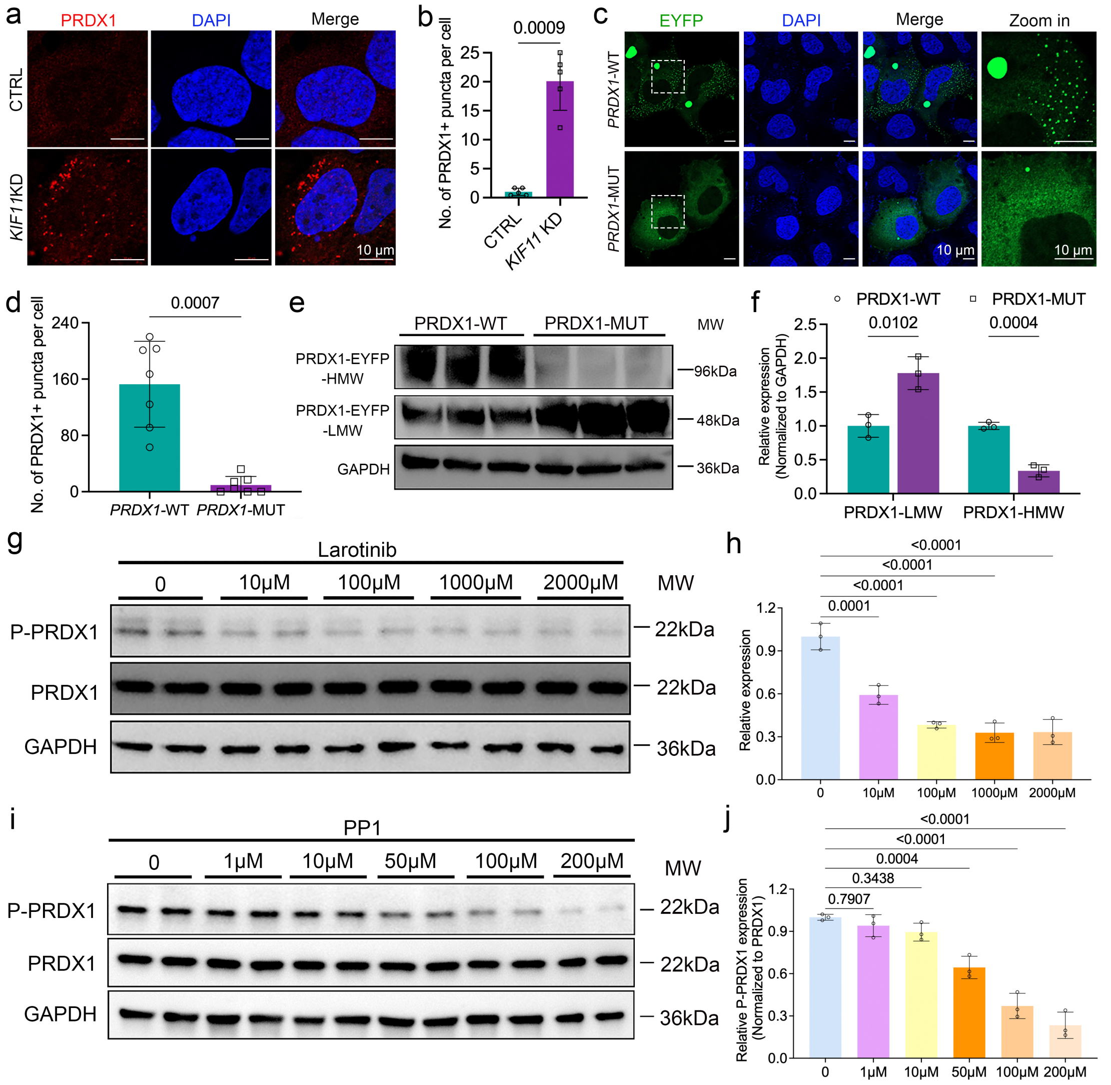

Supplementary Fig. 10 Loss of KIF11 promotes PRDX1 LLPS and Src-mediated phosphorylation. a Representative images of the CTRL and *KIF11* KD HEK293T cells stained with anti-PRDX1 (red) and DAPI (blue). Scale bars, 10 μm. **b** Quantification of PRDX1 puncta numbers per cell in CTRL and *KIF11* KD HEK293T cells. *n* = 5. **c** Representative images of HEK293T cells overexpressing wild-type or Y194Q-mutant PRDX1. Dotted boxes, zoomed-in regions. Green, EYFP; blue, DAPI. Scale bars, 10 μm. **d** Quantification of the puncta numbers per cell in HEK293T cells overexpressing wild-type or Y194Q-mutant PRDX1. *n* = 7. **e** Native-PAGE immunoblotting of wild-type and Y194Q-mutant PRDX1 overexpressed in HEK293T cells. **f** Relative quantification of the HWM and LWM Y194Q-mutant PRDX1 in HEK293T cells, normalized to HWM and LWM wild-type PRDX1, respectively. *n* = 3. **g**, **h** Western blot (**g**) and quantification (**h**) of phosphorylated PRDX1-Tyr194 in *KIF11* KD HRECs treated with increasing concentrations of larotinib (0, 10, 100, 1000, or 2000 μM). *n* = 3. **i**, **j** Western blot (**i**) and quantification (**j**) of phosphorylated PRDX1-Tyr194 in *KIF11* KD HRECs treated with increasing concentrations of PP1 (0, 1, 10, 50, 100, or 200

μM). *n* = 3. Data are presented as mean ± SD. *n* represents independent biological replicates. Statistical significance was determined using a two-tailed Student's t-test (**f**), two-tailed Welch's t-test (**b**, **d**), or one-way ANOVA with Tukey's multiple comparisons test (**h**, **j**). Source data are provided as a Source Data file.

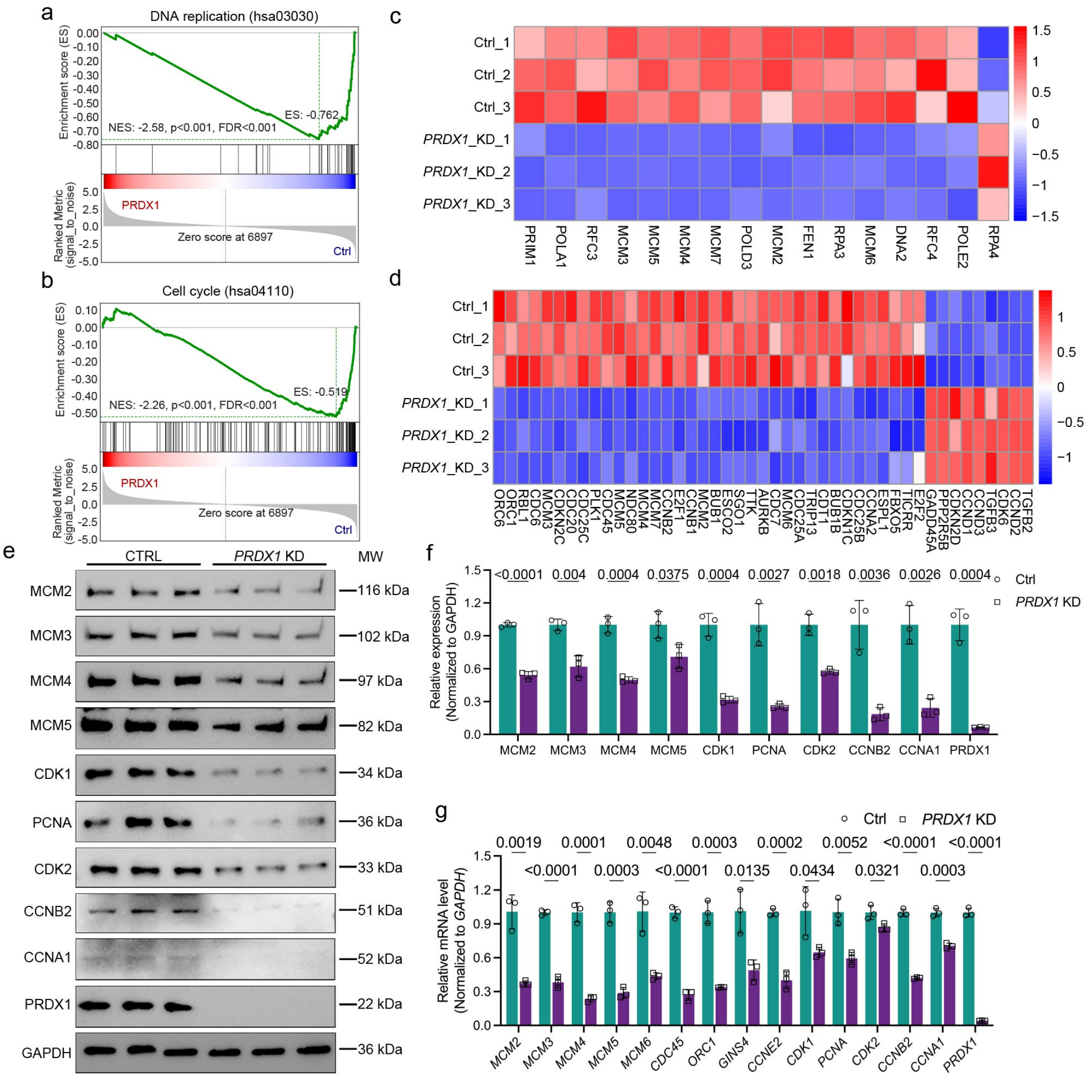

Supplementary Fig. 11 Loss of PRDX1 leads to transcription regulation patterns resembling that of *KIF11*-depleted HRECs. a, **b** GSEA enrichment plots of the KEGG DNA replication (**a**) and cell cycle (**b**) signaling gene sets in CTRL and *PRDX1* KD HRECs. **c**, **d** Heatmaps of the KEGG DNA replication (**c**) and cell cycle (**d**) signaling gene sets for the CTRL and *PRDX1* KD HRECs. **e-g** Western blot analysis (**e**), quantification (**f**), and relative mRNA levels (**g**) of core DNA replication- and cell cycle-related genes in CTRL and *PRDX1* KD HRECs. $n = 3$. Data are presented as mean ± SD. n represents independent biological replicates. Statistical significance was determined using a two-tailed Student's t-test (**f**, **g**). Source data are provided as a Source Data file.

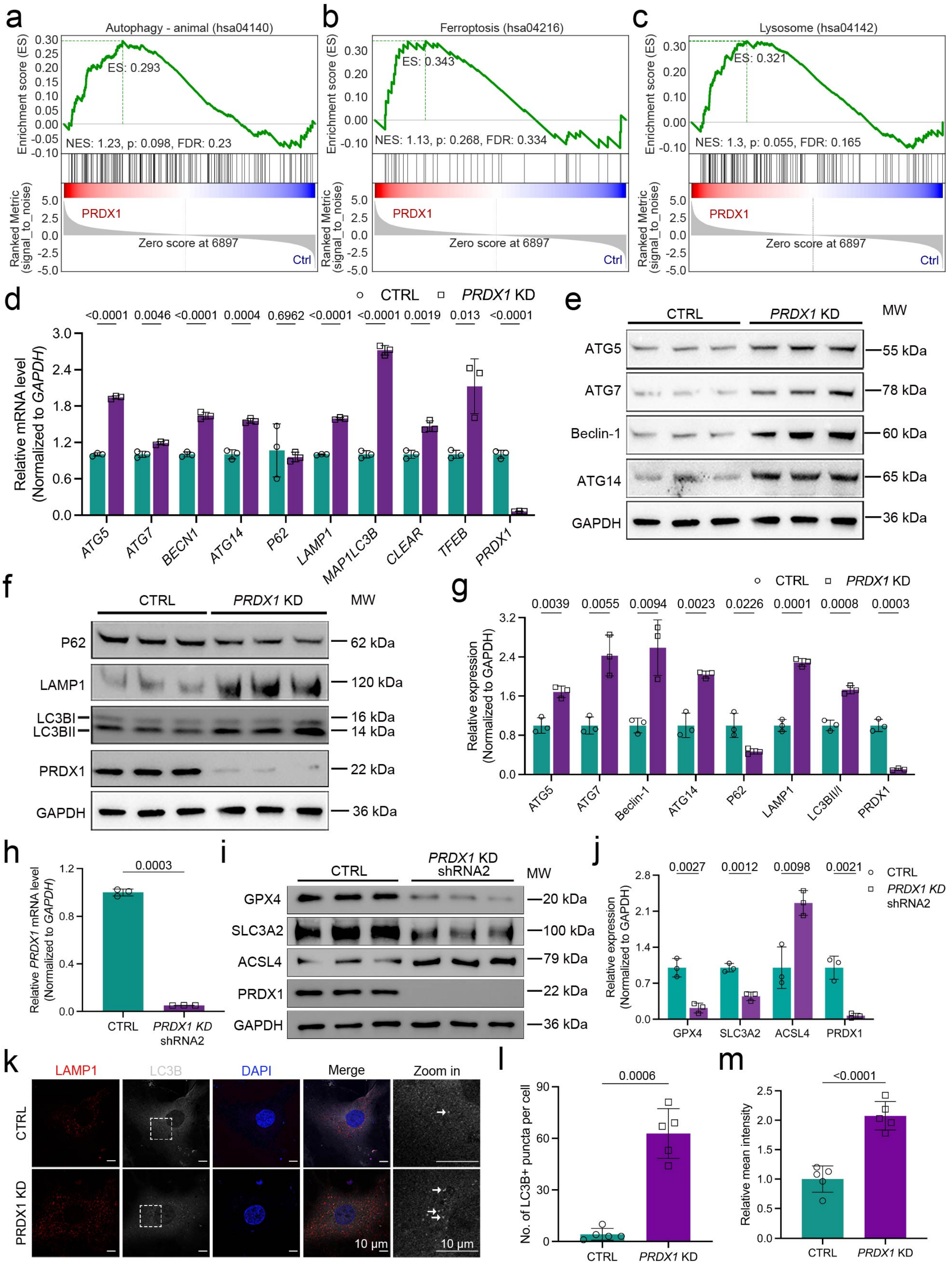

Supplementary Fig. 12 PRDX1 deficiency induces cellular phenotypes associated with autophagy. a-c GSEA enrichment plots of the KEGG autophagy (**a**), ferroptosis (**b**), and lysosome (**c**) signaling gene sets in CTRL and *PRDX1* KD HRECs. **d** Relative mRNA levels of the autophagy- and lysosome-related gene in CTRL and *PRDX1* KD HRECs. n = 3. **e-g** Western blot (**e, f**) and quantification (**g**) of core autophagy- and lysosome-related genes in CTRL and *PRDX1* KD HRECs. n = 3. **h** Relative mRNA levels of *PRDX1* in CTRL versus *PRDX1* KD HRECs using an independent shRNA. n

= 3. **i**, **j** Western blot (**i**) and quantification (**j**) of PRDX1 and core proteins involved in ferroptosis in CTRL and *PRDX1* KD HRECs using an independent shRNA (shRNA2). *n* = 3. **k** Representative immunofluorescent images of CTRL and *PRDX1* KD HRECs. Dotted boxes, magnified regions. Red, LAMP1; white, LC3B; blue, DAPI. Scale bars, 10 μm. **l** Quantification of LC3B puncta numbers per cell in CTRL and *PRDX1* KD HRECs. *n* = 5. **m** Quantification of LAMP1 immunofluorescence intensity in CTRL and *PRDX1* KD HRECs. *n* = 5. Data are presented as mean ± SD. *n* represents independent biological replicates. Statistical significance was determined using a two-tailed Student's t-test (**d**, **g**, **j**, **m**) or two-tailed Welch's t-test (**h**, **l**). Source data are provided as a Source Data file.

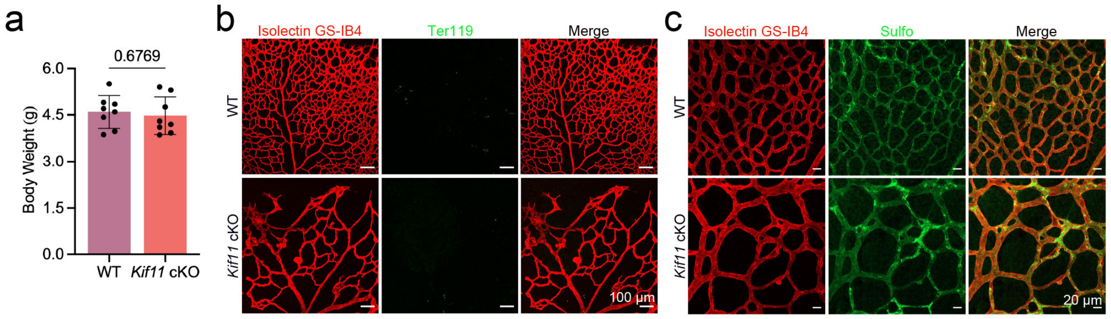

Supplementary Fig. 13 Assessment of body weight and vascular leakage in WT and *Kif11* cKO mice. a Quantification of body weight of P7 WT and *Kif11* cKO mice. n = 8. **b**, **c** Leakage assessment of P7 WT and *Kif11* cKO retinas using anti-Ter119 (green, **b**) or Sulfo-NHS-biotin (green, **c**). Red, Isolectin GS-IB4. Scale bars, 100 μm (**b**) and 20 μm (**c**). Data are presented as mean ± SD. n represents the number of mice per group. Statistical significance was determined using one-way ANOVA with Tukey's multiple comparisons test. Source data are provided as a Source Data file.

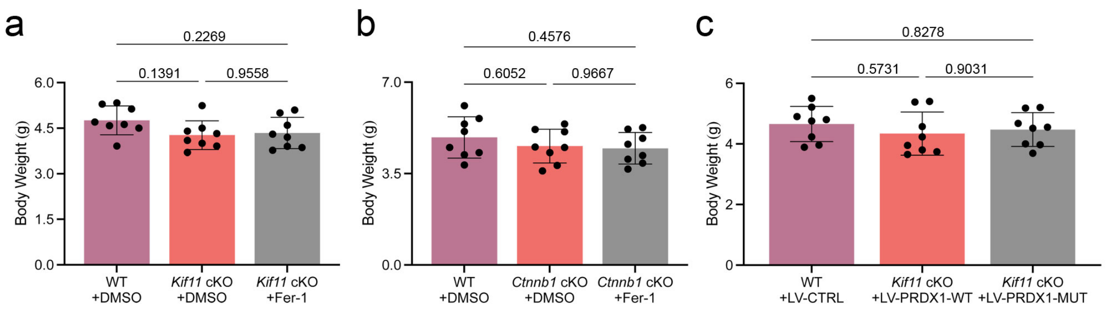

Supplementary Fig. 14 Body weight of mice in Fer-1 and PRDX1-expressing lentivirus rescue experiments. a Quantification of body weight of P7 DMSO-treated WT and DMSO- or Fer-1-treated *Kif11* cKO mice. *n* = 8 mice per group. **b** Quantification of body weight of P7 DMSO-treated WT and DMSO- or Fer-1-treated *Ctnnb1* cKO mice. *n* = 8 mice per group. **c** Quantification of bodyweight of P7 LV-CTRL-treated WT and LV-CTRL-, LV-PRDX1-WT-, or LV-PRDX1-MUT-treated *Kif11* cKO mice. *n* = 8 mice per group. ns: not significant. Data are presented as mean ± SD. *n* represents the number of mice per group. Statistical significance was determined using one-way ANOVA with Tukey's multiple comparisons test. Source data are provided as a Source Data file.

Supplementary Tables 1–6

Supplementary Table 1 Pathogenicity prediction by MutationTaster.

Variants	Prediction
c.466_469del (p.Val156LysfsTer38)	Deleterious
c.1387dupA (p.Thr463AsnfsTer10)	Deleterious
c.2342delA (p.Gln781ArgfsTer18)	Deleterious
c.2579C>A (p.Ser860Ter)	Deleterious
c.2738del (p.Leu913ArgfsTer5)	Deleterious
c.2906_2907del (p.Lys969ArgfsTer19)	Deleterious

Supplementary Table 2 Primers for mouse genotyping.

Primer name	Primers (5'-3')
Pdgfb-iCre-ER-F	GCCGCCGGGATCACTCTCG
Pdgfb-iCre-ER-R	CCAGCCGCCGTCGCAACTC
Kif11$^{\mathrm{loxp/loxp}}$-F	AAAAGCACTGATTGGTTGGTTGGT
Kif11$^{\mathrm{loxp/loxp}}$-R	CCTAGCAAGATATCCAAGGTCAACT
Ctnnb1^{flox}-F	GGCTACTCAAGGTTTGTGATTCAG
Ctnnb1^{flox}-R	AGTCCCAGCAGTACAACGAGC
Tspan12-F1	GGCCAGGATCAAGTTAGAAGAAAT
Tspan12-R1	AAGAAGGCTGCTGAGATTTTCTTGAC
Tspan12-R2	CTCCAAATTGGCATCCTTCAAAAG

Supplementary Table 3 Primers for Sanger sequencing.

Primer name	Primers (5'-3')
KIF11-c.466_469del-F	TTGTTTTGTTTTAACTTGCTTTGC
KIF11-c.466_469del-R	TTATCACTCCTCTCTGACCAAAA
KIF11-c.1387dupA-F	CCGGGTAACTTCTTACAACTTTTG
KIF11-c.1387dupA-R	TCAAAACTCCAGGAGCAGGA
KIF11-c.2342delA-F	GTGAAAAGACAGTGGCCAAG
KIF11-c.2342delA-R	TTATTCTCCCCAAAGTAAGTTCCA
KIF11-c.2579C>A-F	CTGGGAGGTCAAGGCTGTAG
KIF11-c.2579C>A-R	AGGCAATGGCAGGACCTAAT
KIF11-c.2738del-F	TTGTAAGCCAATGTTGTGAGG
KIF11-c.2738del-R	TGTCCTTTTACAAACAGTGCTAGG
KIF11-c.2906_2907del-F	TGAAAACAGTAAAGTCTGTCATCAAA
KIF11-c.2906_2907del-R	AAAAGAAACAAGCCTAAGGGCTA

Supplementary Table 4 shRNA sequences for gene knockdown.

Targeted gene name	Sequence (5’-3’)
FZD4	AGCCAGCTGCAGTTCTTCCTT
LRP5	TGGCCAACCTCAATGGCACAT
TSPAN12	GCAAGCTAACACATTGTCTTA
CTNNB1	TTGGAATGAGACTGCTGAT
KIF11	TTGAATAAGCCTGAAGTGAAT
PRDX1	GATGAGACTTTGAGACTAGTT
Non-targeting Control shRNA	TTCTCCGAACGTGTCACGT

Supplementary Table 5 Antibodies for western blot analysis and immunofluorescence staining.

Antibodies	Catalog	Brand	Dilution	Manufacturer websites
rabbit anti-KIF11	23333-1-AP	Proteintech	1:1000	https://www.ptgcn.com/products/KIF11-Antibody-23333-1-AP.htm
rabbit anti-LRP5	5731S	Cell Signaling Technology	1:1000	https://www.cellsignal.cn/products/primary-antibodies/lrp5-d80f2-rabbit-monoclonal-antibody/5731
rabbit anti-FZD4	A8161	Abclonal	1:1000	https://abclonal.com.cn/catalog/A8161
rabbit anti-TSPAN12	12812-1-AP	Proteintech	1:1000	https://www.ptgcn.com/products/TSPAN12-Antibody-12812-1-AP.htm
rabbit anti-CTNNB1	8480S	Cell Signaling Technology	1:1000	https://www.cellsignal.cn/products/primary-antibodies/beta-catenin-d10a8-rabbit-monoclonal-antibody/8480
rabbit anti-β-actin	AC026	Abclonal	1:1000	https://abclonal.com.cn/catalog/AC026
rabbit anti-GAPDH	A19056	Abclonal	1:1000	https://abclonal.com.cn/catalog/A19056
rabbit anti-FLAG	20543-1-AP	Proteintech	1:1000	https://www.ptgcn.com/products/Flag-Tag-Antibody-20543-1-AP.htm
rabbit anti-MCM2	PTM-5074	PTM BIO	1:1000	https://www.ptmbio.com/products/anti-mcm2-rabbit-mab/PTM-5074.htm

rabbit anti-MCM3	PTM-5733	PTM BIO	1:1000	https://www.ptmbio.com/products/anti-mcm3-rabbit-mab/PTM-5733.htm
rabbit anti-MCM4	13043-1-AP	Proteintech	1:1000	https://www.ptgcn.com/products/MCM4-Antibody-13043-1-AP.htm
mouse anti-MCM5	PTM-5839	PTM BIO	1:1000	https://www.ptmbio.com/products/anti-mcm5-rabbit-mab/PTM-5839.htm
rabbit anti-CDK1	T55176	Abmart	1:1000	https://www.ab-mart.com.cn/page.aspx?node=%2077%20&id=%201463
rabbit anti-PCNA	10205-2-AP	Proteintech	1:1000	https://www.ptgcn.com/products/PCNA-Antibody-10205-2-AP.htm
rabbit anti-CDK2	T55020	Abmart	1:1000	https://www.ab-mart.com.cn/page.aspx?node=%2077%20&id=%201324
rabbit anti-CCNB2	21644-1-AP	Proteintech	1:1000	https://www.ptgcn.com/products/CCNB2-Antibody-21644-1-AP.htm
rabbit anti-CCNA1	13295-1-AP	Proteintech	1:1000	https://www.ptgcn.com/products/CCNA1-Antibody-13295-1-AP.htm
rabbit anti-ATG5	PTM-5868	PTM BIO	1:1000	https://www.ptmbio.com/products/anti-atg5-rabbit-mab/PTM-5868.htm

rabbit anti-ATG7	PTM-6267	PTM BIO	1:1000	https://www.ptmbio.com/products/anti-atg7-rabbit-mab/PTM-6267.htm
rabbit anti-Beclin-1	3495S	Cell Signaling Technology	1:1000	https://www.cellsignal.cn/products/primary-antibodies/beclin-1-d40c5-rabbit-monoclonal-antibody/3495
mouse anti-ATG14	96752S	Cell Signaling Technology	1:1000	https://www.cellsignal.cn/products/primary-antibodies/atg14-d1a1n-rabbit-monoclonal-antibody/96752
rabbit anti-P62	PTM-6434	PTM BIO	1:1000	https://www.ptmbio.com/products/anti-sqstm1-p62-rabbit-mab/PTM-6434.htm
mouse anti-LAMP1	PTM-5775	PTM BIO	1:1000 (WB) 1:100 (IF)	https://www.ptmbio.com/products/anti-lamp1-mouse-mab/PTM-5775.htm
mouse anti-LC3B	83506S	Cell Signaling Technology	1:1000 (WB)	https://www.cellsignal.cn/products/primary-antibodies/lc3b-e5q2k-mouse-monoclonal-antibody/83506
rabbit anti-LC3B	14600-1-AP	Proteintech	1:100 (IF)	https://www.ptgcn.com/products/MAP1LC3B-Antibody-14600-1-AP.htm
rabbit anti-xCT	12691T	Cell Signaling Technology	1:1000	https://www.cellsignal.cn/products/primary-antibodies/xct-slc7a11-d2m7a-rabbit-monoclonal-antibody/12691

rabbit anti-PCBP1	A19276	Abclonal	1:1000	https://abclonal.com.cn/catalog/A19276
rabbit anti-GPX4	52455T	Cell Signaling Technology	1:1000	https://www.cellsignal.cn/products/primary-antibodies/gpx4-antibody/52455
rabbit anti-SLC3A2	47213T	Cell Signaling Technology	1:1000	https://www.cellsignal.cn/products/primary-antibodies/4f2hc-slc3a2-d3f9d-rabbit-monoclonal-antibody/47213
rabbit anti-ACSL4	A20414	Abclonal	1:1000	https://abclonal.com.cn/catalog/A20414
rabbit anti-HO-1	A19062	Abclonal	1:1000	https://abclonal.com.cn/catalog/A19062
anti-FSP1	20886-1-AP	Proteintech	1:1000	https://www.ptgcn.com/products/AIFM2-Antibody-20886-1-AP.htm
anti-DHODH	14877-1-AP	Proteintech	1:1000	https://www.ptgcn.com/products/DHODH-Antibody-14877-1-AP.htm
rabbit anti-PRDX1	15816-1-AP	Proteintech	1:1000 (WB) 1:100 (IF)	https://www.ptgcn.com/products/PRDX1-Antibody-15816-1-AP.htm
rabbit anti-CLK3	28037-1-AP	Proteintech	1:1000	https://www.ptgcn.com/products/CLK3-Antibody-28037-1-AP.htm
rabbit anti-IK	YT2297	Immunoway	1:1000	https://www.immunoway.com/products/primary-antibodies/YT2297-IK-Rabbit-pAb.html

rabbit anti-SEC16A	20025-1-AP	Proteintech	1:1000	https://www.ptgcn.com/products/SEC16A-Antibody-20025-1-AP.htm
rabbit anti-MPRIP	20040-1-AP	Proteintech	1:1000	https://www.ptgcn.com/products/MPRIP-Antibody-20040-1-AP.htm
rabbit anti-EIF5A	11309-1-AP	Proteintech	1:1000	https://www.ptgcn.com/products/EIF5A-Antibody-11309-1-AP.htm
rat anti-HA	11867423001	Roche	1:1000	https://www.sigmaaldrich.cn/CN/zh/product/roche/roahaha
anti-Ter119	553671	BD Biosciences	1:100	https://www.bdbiosciences.com/en-us/products/reagents/functional-cell-based-reagents/purified-rat-anti-mouse-ter-119-erythroid-cells.553671?tab=product_details
rabbit anti-phos-PRDX1-Tyr194	14041S	Cell Signaling Technology	1:1000	https://www.cellsignal.cn/products/primary-antibodies/phospho-prdx1-tyr194-d1t9c-rabbit-monoclonal-antibody/14041
rabbit anti-GFP	50430-2-AP	Proteintech	1:1000	https://www.ptgcn.com/products/eGFP-Antibody-50430-2-AP.htm
Isolectin GS-IB4 Alexa Fluor™	I21413	Invitrogen	1:200	https://www.thermofisher.cn/order/catalog/product/I21413?adobe_mc=MCMID%7C244014281137244161005185244262312353 8

594 Conjugate				9%7CMCAID%3D3423F6676A295728-40000FA361BF316C%7CMCORGID%3D5B135A0C5370E6B40A490D44%40AdobeOrg%7CTS=1614293705
anti-mouse IgG (H+L), Alexa Fluor Plus 594	A-21203	Invitrogen	1:200	https://www.thermofisher.cn/cn/zh/antibody/product/Donkey-anti-Mouse-IgG-H-L-Highly-Cross-Adsorbed-Secondary-Antibody-Polyclonal/A-21203
anti-rat IgG (H+L), Alexa Fluor 488	A-21208	Invitrogen	1:200	https://www.thermofisher.cn/cn/zh/antibody/product/Donkey-anti-Rat-IgG-H-L-Highly-Cross-Adsorbed-Secondary-Antibody-Polyclonal/A-21208?adobe_mc=MCMID%7C24401428113724416100518524426231235389%7CMCAID%3D3423F6676A295728-40000FA361BF316C%7CMCORGID%3D5B135A0C5370E6B40A490D44%40AdobeOrg%7CTS=1614293705
anti-rabbit IgG (H+L), Alexa	A-31573	Invitrogen	1:200	https://www.thermofisher.cn/cn/zh/antibody/product/Donkey-anti-Rabbit-IgG-H-L-Highly-

Fluor Plus 647				Cross-Adsorbed-Secondary-Antibody-Polyclonal/A-31573
Hoechst	C1017	Beyotime	1:200	https://www.beyotime.com/product/C1017.htm
DAPI	4083	Cell Signaling Technology	1:200	https://www.cellsignal.cn/products/buffers-dyes/dapi/4083

Supplementary Table 6 Primers for RT-qPCR.

Primer name	Primers (5'-3')
FZD4-F	TCTTCTCTGTGCACATTGGC
FZD4-R	GACAACTTTCACACCGCTCA
TSPAN12-F	ACGCCACAAGCCAGTTCTAC
TSPAN12-R	TGTTTCCTTATCATTGTGGGG
LRP5-F	GAGATCCTCCGTAGGTCCGT
LRP5-R	CCAAGCGAGCCTTTCTACAC
CTNNB1-F	AGGTCTGAGGAGCAGCTTCA
CTNNB1-R	ATTGTCCACGCTGGATTTTC
PRDX1-F	CACGGAGATCATTGCTTTCA
PRDX1-R	CGGGTCTGATACCAAAGGAA
KIF11-F	CACAACAAGGATGAAGTCTATCAAA
KIF11-R	CAAGCTCTTCTCCATCAATCG
ATG5-F	CAGATGGACAGTTGCACACA
ATG5-R	TCCGGGTAGCTCAGATGTTC
ATG7-F	TTAGCCCAGTACCCTGGATG
ATG7-R	CTTCGAAGATGATGCTGTGG
BECN1-F	GCTGGATGATGAGCTGAAGA
BECN1-R	CGACCCAGCCTGAAGTTATT
ATG14-F	AACCCTGCCTACACCATCAG
ATG14-R	TGCATTCAGTTTCTTCACTGC
P62-F	CTCCAGTCCCTACAGATGCC
P62-R	TCAGAGAAGCCCATGGACAG
LAMP1-F	CACGCTGTGAACAAGACAGG
LAMP1-R	TGTTGGGGTTGATGTTGAGA
MAP1LC3B-F	CGATACAAGGGTGAGAAGCAG
MAP1LC3B-R	CTGTGTCCGTTCACCAACAG
CLEAR-F	AGAAGCTTCCCCATGGTCTT
CLEAR-R	GTTCTCTCCAAGGCCCAGAT
TFEB-F	AGCAGCCACCTGAATGTGTA
TFEB-R	CTGCATCCTCCGGATGTAAT
GAPDH-F	CTGACTTCAACAGCGACACC
GAPDH-R	TGCTGTAGCCAAATTCGTTG
SLC7A11-F	AGTCTGGGTGGAACTCCTCA
SLC7A11-R	AGCTTGATCGCAAGTTCAGG
PCBP1-F	GGAAGCATCATTGGGAAGAA
PCBP1-R	TCTTCCTCCAGCTTGTCGAT
GPX4-F	CTTCCCGTGTAACCAGTTCG

GPX4-R	TCACGCAGATCTTGCTGAAC
SLC3A2-F	CAGAAGGATGATGTCGCTCA
SLC3A2-R	ACCCCGGTAGTTGGGAGTAA
ACSL4-F	CACCATTGCCATCTTCTGTG
ACSL4-R	CTGCTTCTTTGCCAAGTGTG
HOMX1-F	ATGACACCAAGGACCAGAGC
HOMX1-R	GTGTAAGGACCCATCGGAGA
MCM2-F	CTACCTTTCATTCCGGCGTG
MCM2-R	TGCAGAGAGGTTGTGGATGT
MCM3-F	AACTCTGATTCGACTGGCCA
MCM3-R	TTCTGCTCCTGGTCCTCTTG
MCM4-F	GAGTGCCACCTCTCGTAAAC
MCM4-R	GGTCTTCCCAGTCACTGTCA
MCM5-F	GAGGACCAGGAGATGCTGAG
MCM5-R	TCACTTGAGGCGGTAGAGAA
MCM6-F	GGCCGAGAAGTGCCAGAAACTG
MCM6-R	AGCTCTGGGTGAACTGGGTGAG
CDC45-F	GCACTCCAGATGTCATGCTG
CDC45-R	TTCTTCCTGTCCGAGCTGTC
ORC1-F	GGAAGCCACGTTTCAACAGA
ORC1-R	CATCATCCTGGCTGACGTTG
Gins4-F	AGGTCCCCGAGTTCCTGGTTTC
Gins4-R	GACACCGCAAGTAGCTGCTGAG
CCNE2-F	TCTGCATTCTGACTTGGAACC
CCNE2-R	CTCAAGTTTGGAAGCAATGAA
CDK1-F	GGTCAAGTGGTAGCCATGAAA
CDK1-R	CCAGGAGGGATAGAATCCAA
PCNA-F	TTCCTGTGCAAAAGACGGAG
PCNA-R	TCACCGTTGAAGAGAGTGGA
CDK2-F	GCCCTATTCCCTGGAGATTC
CDK2-R	CTTCATCCAGGGGAGGTACA
CCNB2-F	TCAACCCACCAAAACAACAA
CCNB2-R	AGGGTTCTCCCAATCTTCGT
CCNA1-F	ACCCCAAGAGTGGAGTTGTG
CCNA1-R	CCCCTGCTCTAGTTCATCCA

Uncropped scans of blots and gels in Supplementary Figures
Supplementary Figure 4

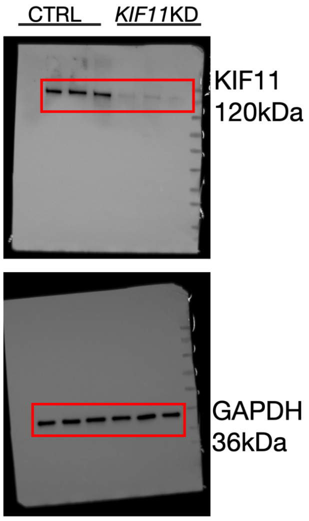

Supplementary Figure 6

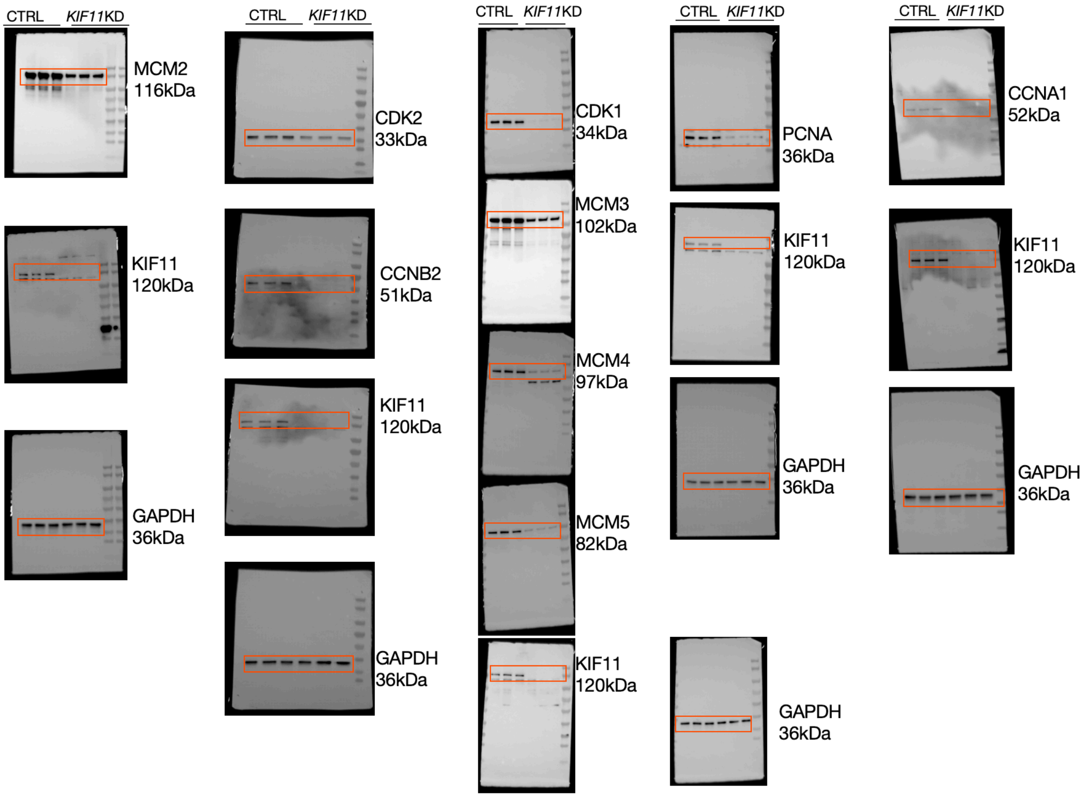

Supplementary Figure 7

Supplementary Figure 7b

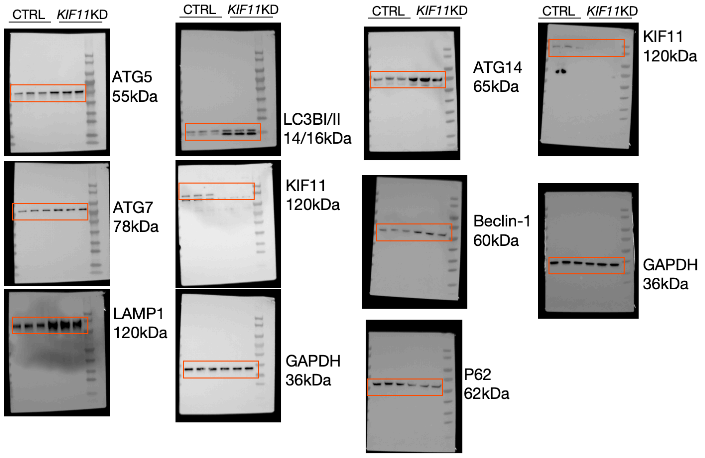

Supplementary Figure 7i

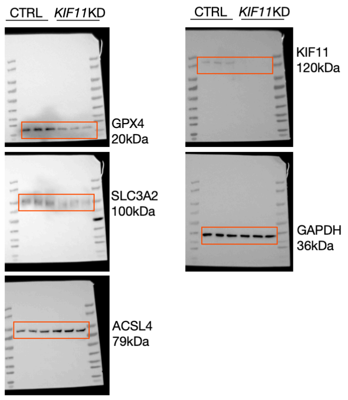

Supplementary Figure 8

Supplementary Figure 8e

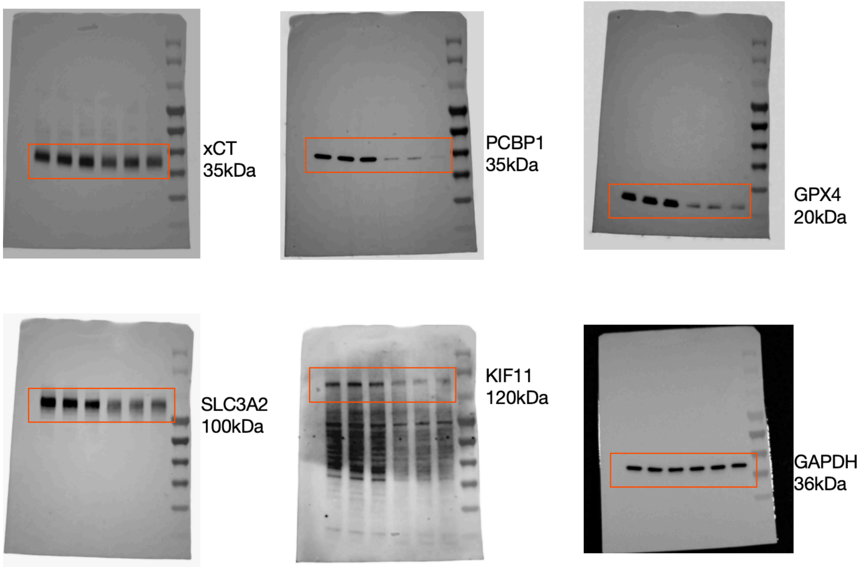

Supplementary Figure 9

Supplementary Figure 9a

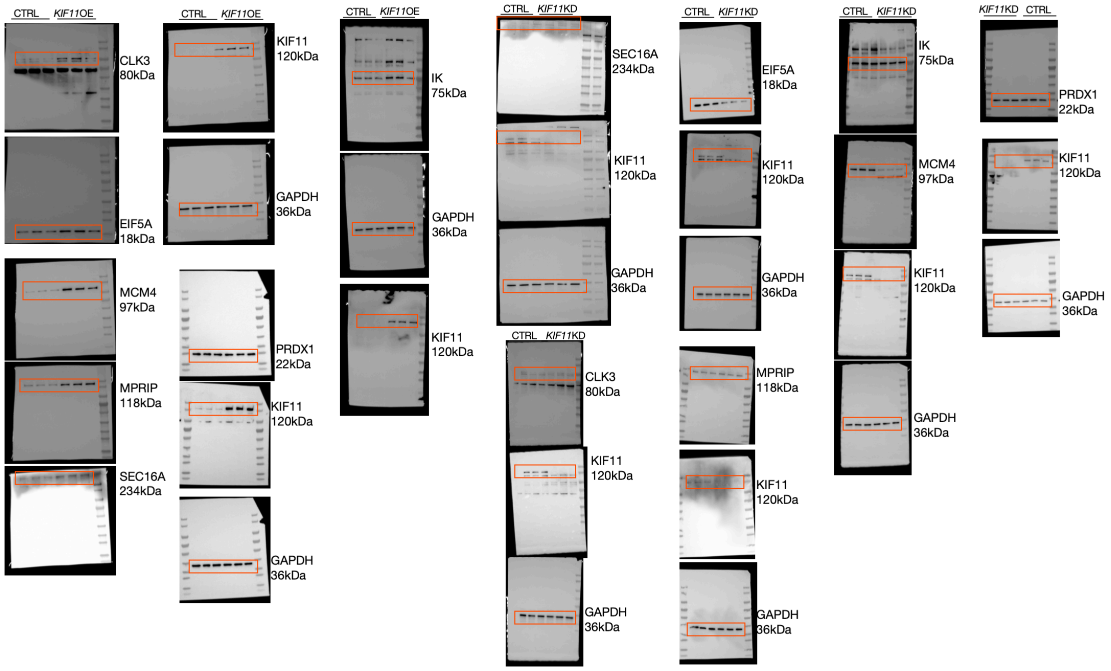

Supplementary Figure 10

Supplementary Figure 10e

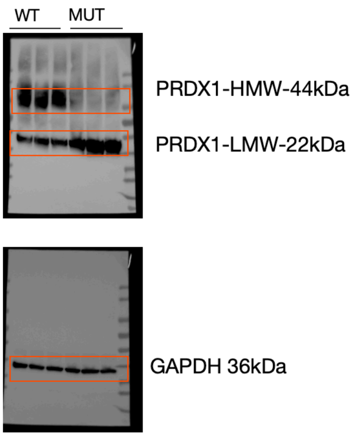

Supplementary Figure 10g and 10i

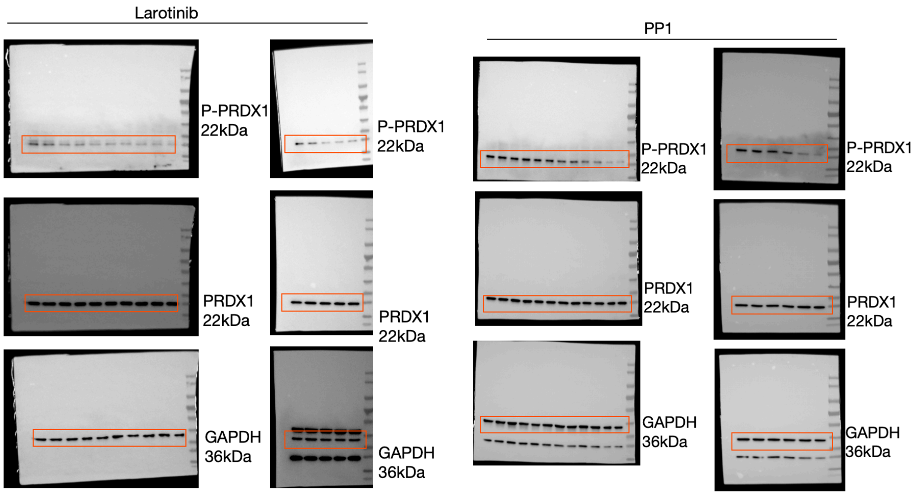

Supplementary Figure 11

Supplementary Figure 11e

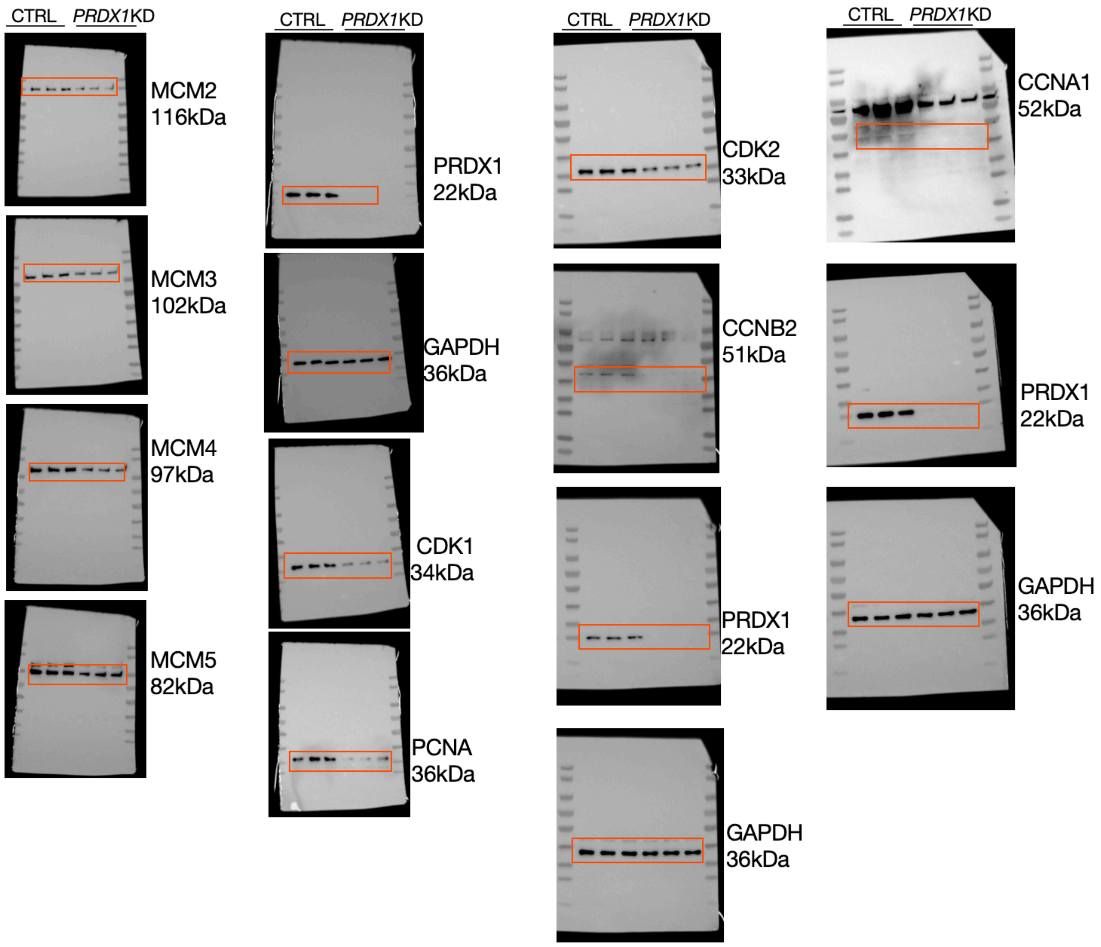

Supplementary Figure 12

Supplementary Figure 12e

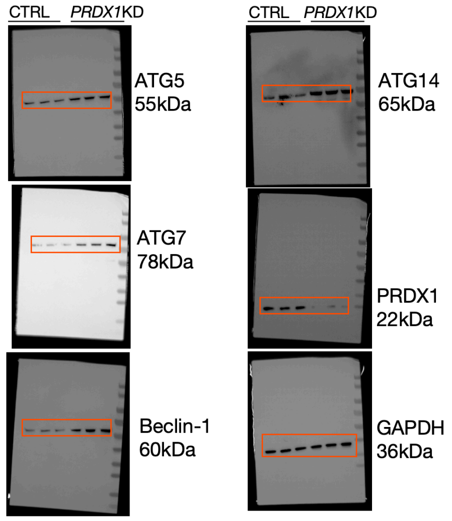

Supplementary Figure 12f

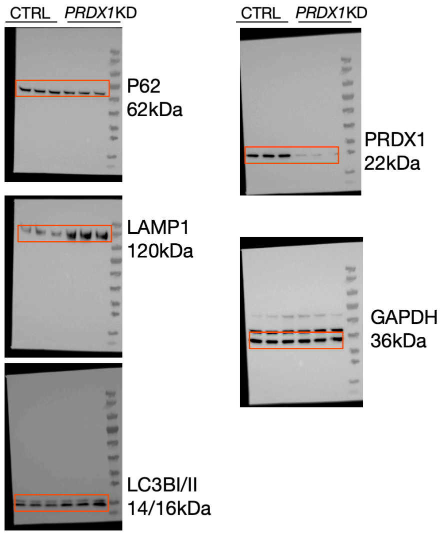

Supplementary Figure 12i

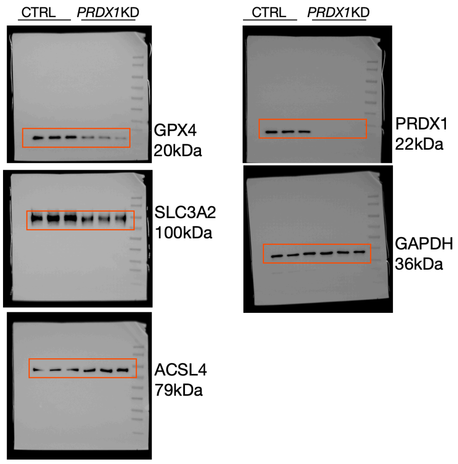

Supplement: Supplementary file 1 — Supplementary Information [file 41467_2026_71009_MOESM1_ESM.pdf]
