# Supplementary material for: KIF11 prevents retinal endothelial ferroptosis in familial exudative vitreoretinopathy by inhibiting phosphorylation-driven PRDX1 phase separation
Source: Nat Commun. 2026 Mar 24;17:4360. doi: 10.1038/s41467-026-71009-7 (PMC13172007; doi:10.1038/s41467-026-71009-7)
Supplement: Supplementary file 2 — Description Of Additional Supplementary File [file 41467_2026_71009_MOESM2_ESM.pdf]

Description of Additional Supplementary Files

Supplementary Data 1: FPKM values for bulk RNA-seq.
Supplementary Data 2: Venn analysis for commonly regulated genes in *CTNNB1* KD, *FZD4* KD, *TSPAN12* KD, and *LRP5* KD HRECs.
