# Supplementary material for: KIF11 prevents retinal endothelial ferroptosis in familial exudative vitreoretinopathy by inhibiting phosphorylation-driven PRDX1 phase separation
Source: Nat Commun. 2026 Mar 24;17:4360. doi: 10.1038/s41467-026-71009-7 (PMC13172007; doi:10.1038/s41467-026-71009-7)
Supplement: Supplementary file 5 — Reporting Summary [file 41467_2026_71009_MOESM5_ESM.pdf]

nature portfolio

Corresponding author(s): Shujin Li and Zhenglin Yang

Last updated by author(s): Sep 5, 2025

Reporting Summary

Nature Portfolio wishes to improve the reproducibility of the work that we publish. This form provides structure for consistency and transparency in reporting. For further information on Nature Portfolio policies, see our Editorial Policies and the Editorial Policy Checklist.

Statistics

For all statistical analyses, confirm that the following items are present in the figure legend, table legend, main text, or Methods section.

n/a	Confirmed	
☐	☒	The exact sample size (*n*) for each experimental group/condition, given as a discrete number and unit of measurement
☐	☒	A statement on whether measurements were taken from distinct samples or whether the same sample was measured repeatedly
☐	☒	The statistical test(s) used AND whether they are one- or two-sided<br>*Only common tests should be described solely by name; describe more complex techniques in the Methods section.*
☒	☐	A description of all covariates tested
☐	☒	A description of any assumptions or corrections, such as tests of normality and adjustment for multiple comparisons
☐	☒	A full description of the statistical parameters including central tendency (e.g. means) or other basic estimates (e.g. regression coefficient) AND variation (e.g. standard deviation) or associated estimates of uncertainty (e.g. confidence intervals)
☐	☒	For null hypothesis testing, the test statistic (e.g. *F*, *t*, *r*) with confidence intervals, effect sizes, degrees of freedom and *P* value noted<br>*Give P values as exact values whenever suitable.*
☒	☐	For Bayesian analysis, information on the choice of priors and Markov chain Monte Carlo settings
☒	☐	For hierarchical and complex designs, identification of the appropriate level for tests and full reporting of outcomes
☐	☒	Estimates of effect sizes (e.g. Cohen's *d*, Pearson's *r*), indicating how they were calculated

Our web collection on statistics for biologists contains articles on many of the points above.

Software and code

Policy information about availability of computer code

Data collection

Targeted gene sequencing was performed on Illumina Nextseq 500 platform (Illumina) by MyGenostics.
Bulk RNA-sequencing was performed using the Illumina NovaSeqXplus or NovaSeq 6000 system (150 bp × 2, Shanghai BIOZERON Co., Ltd).
Single-RNA sequencing was performed on an Illumina NovaSeq 6000 platform (PE150) by OE Biotech (Shanghai, China).
The LC-MS/MS analysis was conducted on an Orbitrap Exploris 480 or a timsTOF Pro mass spectrometer (Bruker) coupled to an Evosep One liquid chromatography system (Denmark) by Shanghai GENECHEM Co., Ltd and Applied Protein Technology.
LC-MS/MS analysis of phospholipid peroxidation: Mass spectrometric detection was performed on a QTRAP® 6500+ system (SCIEX, UK) equipped with an electrospray ionization (ESI) source. The analysis was conducted in multiple reaction monitoring (MRM) mode, monitoring transitions from precursor ions to product ions. The optimized ion source parameters were as follows: ion spray voltage (IS), –4500 V; source temperature (TEM), 500 °C; curtain gas (CUR), 35 psi; nebulizer gas (GS1), 45 psi; auxiliary gas (GS2), 55 psi. The compound-dependent parameters were set as declustering potential (DP), –50 V; entrance potential (EP), –10 V; and collision energy (CE), –38 V.
LSM900 confocal microscope (Zeiss).

Data analysis

Single-RNA sequencing analysis: Cell Ranger software (version 9.0.0, 10× Genomics), Seurat package (v4.0.0) , Harmony R package (v1.0).
Targeted gene sequencing: Cutadapt (http://code.google.com/p/cutadapt/), BWA (http://bio-bwa.sourceforge.net/), Picard tools (http://broadinstitute.github.io/picard/), HaplotypeCaller in GATK (https://software.broadinstitute.org/gatk/), ANNOVAR (http://annovar.openbioinformatics.org/en/latest/).
The MS data were analyzed by MaxQuant (version 1.6.14).
Bulk RNA-sequencing: Trimmomatic (v0.39), HISAT2 (version 2.1.0), Qualimap (version 2.2.1), HTSeq (version 0.11.1), edgeR package (version 3.34.0), sva R package (v3.44.0),WGCNA R packages (version 1.71), Cytoscape (version 3.10.1), clusterProfiler (version 3.14.3), GSEABase package (version 1.44.0).
ATAC-seq analysis: trimGalore (version 1.18), Bowtie2 alignment tool (version 2.3.5.1), MACS2 (version 2.2.7.1), Deeptools (version 3.4.3),

Proteomics: Limma (version 3.40.6), MaxQuant software (version 1.5.2.8).
Image J (version 2.16.0)
ZEN 2.6 (Blue Edition).
GraphPad Prism 9.0.

For manuscripts utilizing custom algorithms or software that are central to the research but not yet described in published literature, software must be made available to editors and reviewers. We strongly encourage code deposition in a community repository (e.g. GitHub). See the Nature Portfolio guidelines for submitting code & software for further information.

Data

Policy information about availability of data

All manuscripts must include a data availability statement. This statement should provide the following information, where applicable:

- Accession codes, unique identifiers, or web links for publicly available datasets
- A description of any restrictions on data availability
- For clinical datasets or third party data, please ensure that the statement adheres to our policy

The data supporting the findings from this study are available within the manuscript and its supplementary information. The raw targeted sequencing data generated in this study have been deposited in the Genome Sequence Archive in National Genomics Data Center, China National Center for Bioinformation, Beijing Institute of Genomics, Chinese Academy of Sciences, under accession number HRA009047 (https://ngdc.cncb.ac.cn/gsa-human/browse/HRA009047). Researchers wishing to access the data must submit a formal request to the corresponding author, Shujin Li (lishujin91@126.com). The request should include a brief research proposal and a signed Data Use Agreement (DUA). We will respond to all access requests within 4 weeks. The data are restricted to academic use only and must not be used for re-identification of participants or shared with third parties without prior authorization. The raw data of bulk RNA-seq from CTNNB1-, FZD4-, LRP5-, TSPAN12-, KIF11-, and PRDX1-depleted HRECs, along with control HRECs, have been deposited in the Sequence Read Archive (SRA; PRJNA1297355, https://www.ncbi.nlm.nih.gov/bioproject/PRJNA1297355). The processed data for RNA-seq have been deposited in GEO (GSE306946, https://www.ncbi.nlm.nih.gov/geo/query/acc.cgi?acc=GSE306946). FPKM values for bulk RNA-seq were listed in Supplementary Data 1. The raw data of scRNA-seq from P6 WT, P6 Tspan12 KO, and P6 Ctnnb1 cKO mice have been deposited in GEO database (GSE305756, https://www.ncbi.nlm.nih.gov/geo/query/acc.cgi?acc=GSE305756). The mass spectrometry proteomics data of KIF11-binding proteins and KIF11-depleted HRECs have been deposited in the ProteomeXchange Consortium (https://www.ebi.ac.uk/pride/archive/projects) via the PRIDE partner repository, with the dataset identifiers PXD074452 (https://www.ebi.ac.uk/pride/archive/projects/PXD074452) and PXD074456 (https://www.ebi.ac.uk/pride/archive/projects/PXD074456), respectively. The processed data of targeted phospholipid peroxidation have been deposited to MetaboLights (https://www.ebi.ac.uk/metabolights/MTBLS12912). Source data are provided with this paper.

Research involving human participants, their data, or biological material

Policy information about studies with human participants or human data. See also policy information about sex, gender (identity/presentation), and sexual orientation and race, ethnicity and racism.

Reporting on sex and gender	Both male and female participants were enrolled.
Reporting on race, ethnicity, or other socially relevant groupings	N/A
Population characteristics	N/A
Recruitment	The patients were enrolled and evaluated at the Xinhua Hospital Affiliated with Shanghai Jiaotong University School of Medicine. Written informed consent was obtained from all participants or the legal guardians of minors. The diagnostic criteria for familial exudative vitreoretinopathy (FEVR) primarily consist of the following: (1) full-term birth with normal birth weight and no history of oxygen support; (2) presence of an avascular peripheral retina; (3) displaced or dragged retinal vessels and macula; (4) retinal folds (falciform) or retinal detachment; (5) neovascularization or subretinal exudates.
Ethics oversight	The clinical study complied with the Declaration of Helsinki and received approval from the ethical review boards of Sichuan Provincial People's Hospital (No.465, 2021) and Xinhua Hospital at Shanghai Jiaotong University (XHEC-KJB-2024-013).

Note that full information on the approval of the study protocol must also be provided in the manuscript.

Field-specific reporting

Please select the one below that is the best fit for your research. If you are not sure, read the appropriate sections before making your selection.

☒ Life sciences ☐ Behavioural & social sciences ☐ Ecological, evolutionary & environmental sciences

For a reference copy of the document with all sections, see nature.com/documents/nr-reporting-summary-flat.pdf

Life sciences study design

All studies must disclose on these points even when the disclosure is negative.

Sample size	The sample size was not calculated. A sample size that reflects biological and technological variation was determined. For all studies, sufficient experimental replicates were performed to calculate the mean and standard deviation.

Data exclusions	No samples were excluded from analysis.
Replication	At least 3 independent experimental replicates or mice were used for all analyses.
Randomization	Animals were assigned to experimental groups based on genotype.
Blinding	Data acquisition and quantification were performed by investigators blinded to the experimental groups, while sample allocation was determined based on genotype.

Reporting for specific materials, systems and methods

We require information from authors about some types of materials, experimental systems and methods used in many studies. Here, indicate whether each material, system or method listed is relevant to your study. If you are not sure if a list item applies to your research, read the appropriate section before selecting a response.

Materials & experimental systems

n/a	Involved in the study
☐	☒ Antibodies
☐	☒ Eukaryotic cell lines
☒	☐ Palaeontology and archaeology
☐	☒ Animals and other organisms
☒	☐ Clinical data
☒	☐ Dual use research of concern
☒	☐ Plants

Methods

n/a	Involved in the study
☒	☐ ChIP-seq
☒	☐ Flow cytometry
☒	☐ MRI-based neuroimaging

Antibodies

Antibodies used

rabbit anti-KIF11 23333-1-AP Proteintech 1:1000 https://www.ptgcn.com/products/KIF11-Antibody-23333-1-AP.htm
rabbit anti-LRP5 5731S Cell Signaling Technology 1:1000 https://www.cellsignal.cn/products/primary-antibodies/lrp5-d80f2-rabbit-monoclonal-antibody/5731
rabbit anti-FZD4 A8161 Abclonal 1:1000 https://abclonal.com.cn/catalog/A8161
rabbit anti-TSPAN12 12812-1-AP Proteintech 1:1000 https://www.ptgcn.com/products/TSPAN12-Antibody-12812-1-AP.htm
rabbit anti-CTNNB1 8480S Cell Signaling Technology 1:1000 https://www.cellsignal.cn/products/primary-antibodies/beta-catenin-d10a8-rabbit-monoclonal-antibody/8480
rabbit anti-β-actin AC026 Abclonal 1:1000 https://abclonal.com.cn/catalog/AC026
rabbit anti-GAPDH A19056 Abclonal 1:1000 https://abclonal.com.cn/catalog/A19056
rabbit anti-FLAG 20543-1-AP Proteintech 1:1000 https://www.ptgcn.com/products/Flag-Tag-Antibody-20543-1-AP.htm
rabbit anti-MCM2 PTM-5074 PTM BIO 1:1000 https://www.ptmbio.com/products/anti-mcm2-rabbit-mab/PTM-5074.htm
rabbit anti-MCM3 PTM-5733 PTM BIO 1:1000 https://www.ptmbio.com/products/anti-mcm3-rabbit-mab/PTM-5733.htm
rabbit anti-MCM4 13043-1-AP Proteintech 1:1000 https://www.ptgcn.com/products/MCM4-Antibody-13043-1-AP.htm
mouse anti-MCM5 PTM-5839 PTM BIO 1:1000 https://www.ptmbio.com/products/anti-mcm5-rabbit-mab/PTM-5839.htm
rabbit anti-CDK1 T55176 Abmart 1:1000 https://www.ab-mart.com.cn/page.aspx?node=%2077%20&id=%201463
rabbit anti-PCNA 10205-2-AP Proteintech 1:1000 https://www.ptgcn.com/products/PCNA-Antibody-10205-2-AP.htm
rabbit anti-CDK2 T55020 Abmart 1:1000 https://www.ab-mart.com.cn/page.aspx?node=%2077%20&id=%201324
rabbit anti-CCNB2 21644-1-AP Proteintech 1:1000 https://www.ptgcn.com/products/CCNB2-Antibody-21644-1-AP.htm
rabbit anti-CCNA1 13295-1-AP Proteintech 1:1000 https://www.ptgcn.com/products/CCNA1-Antibody-13295-1-AP.htm
rabbit anti-ATG5 PTM-5868 PTM BIO 1:1000 https://www.ptmbio.com/products/anti-atg5-rabbit-mab/PTM-5868.htm
rabbit anti-ATG7 PTM-6267 PTM BIO 1:1000 https://www.ptmbio.com/products/anti-atg7-rabbit-mab/PTM-6267.htm
rabbit anti-Beclin-1 3495S Cell Signaling Technology 1:1000 https://www.cellsignal.cn/products/primary-antibodies/beclin-1-d40c5-rabbit-monoclonal-antibody/3495
mouse anti-ATG14 96752S Cell Signaling Technology 1:1000 https://www.cellsignal.cn/products/primary-antibodies/atg14-d1a1n-rabbit-monoclonal-antibody/96752
rabbit anti-P62 PTM-6434 PTM BIO 1:1000 https://www.ptmbio.com/products/anti-sqstm1-p62-rabbit-mab/PTM-6434.htm
mouse anti-LAMP1 PTM-5775 PTM BIO 1:1000 (WB)
1:100 (IF) https://www.ptmbio.com/products/anti-lamp1-mouse-mab/PTM-5775.htm
mouse anti-LC3B 83506S Cell Signaling Technology 1:1000 (WB) https://www.cellsignal.cn/products/primary-antibodies/lc3b-e5q2k-mouse-monoclonal-antibody/83506
rabbit anti-LC3B 14600-1-AP Proteintech 1:100 (IF) https://www.ptgcn.com/products/MAP1LC3B-Antibody-14600-1-AP.htm
rabbit anti-xCT 12691T Cell Signaling Technology 1:1000 https://www.cellsignal.cn/products/primary-antibodies/xct-slc7a11-d2m7a-rabbit-monoclonal-antibody/12691
rabbit anti-PCBP1 A19276 Abclonal 1:1000 https://abclonal.com.cn/catalog/A19276
rabbit anti-GPX4 52455T Cell Signaling Technology 1:1000 https://www.cellsignal.cn/products/primary-antibodies/gpx4-antibody/52455
rabbit anti-SLC3A2 47213T Cell Signaling Technology 1:1000 https://www.cellsignal.cn/products/primary-antibodies/4f2hc-slc3a2-d3f9d-rabbit-monoclonal-antibody/47213
rabbit anti-ACSL4 A20414 Abclonal 1:1000 https://abclonal.com.cn/catalog/A20414
rabbit anti-HO-1 A19062 Abclonal 1:1000 https://abclonal.com.cn/catalog/A19062
anti-FSP1 20886-1-AP Proteintech 1:1000 https://www.ptgcn.com/products/AIFM2-Antibody-20886-1-AP.htm

anti-DHODH 14877-1-AP Proteintech 1:1000 https://www.ptgcn.com/products/DHODH-Antibody-14877-1-AP.htm
rabbit anti-PRDX1 15816-1-AP Proteintech 1:1000 (WB)
1:100 (IF) https://www.ptgcn.com/products/PRDX1-Antibody-15816-1-AP.htm
rabbit anti-CLK3 28037-1-AP Proteintech 1:1000 https://www.ptgcn.com/products/CLK3-Antibody-28037-1-AP.htm
rabbit anti-IK YT2297 Immunoway 1:1000 https://www.immunoway.com/products/primary-antibodies/YT2297-IK-Rabbit-pAb.html
rabbit anti-SEC16A 20025-1-AP Proteintech 1:1000 https://www.ptgcn.com/products/SEC16A-Antibody-20025-1-AP.htm
rabbit anti-MPRIP 20040-1-AP Proteintech 1:1000 https://www.ptgcn.com/products/MPRIP-Antibody-20040-1-AP.htm
rabbit anti-EIF5A 11309-1-AP Proteintech 1:1000 https://www.ptgcn.com/products/EIF5A-Antibody-11309-1-AP.htm
rat anti-HA 11867423001 Roche 1:1000 https://www.sigmaaldrich.cn/CN/zh/product/roche/roahaha
anti-Ter119 553671 BD Biosciences 1:100 https://www.bdbiosciences.com/en-us/products/reagents/functional-cell-based-reagents/purified-rat-anti-mouse-ter-119-erythroid-cells.553671?tab=product_details
rabbit anti-phos-PRDX1-Tyr194 14041S Cell Signaling Technology 1:1000 https://www.cellsignal.cn/products/primary-antibodies/phospho-prdx1-tyr194-d1t9c-rabbit-monoclonal-antibody/14041
rabbit anti-GFP 50430-2-AP Proteintech 1:1000 https://www.ptgcn.com/products/eGFP-Antibody-50430-2-AP.htm
Isolectin GS-IB4 Alexa Fluor™ 594 Conjugate I21413 Invitrogen 1:200 https://www.thermofisher.cn/order/catalog/product/I21413?adobe_mc=MCMID%7C24401428113724416100518524426231235389%7CMCAID%3D3423F6676A295728-40000FA361BF316C%7CMCORGID%3D5B135A0C5370E6B40A490D44%40AdobeOrg%7CTS=1614293705
anti-mouse IgG (H+L), Alexa Fluor Plus 594 A-21203 Invitrogen 1:200 https://www.thermofisher.cn/cn/zh/antibody/product/Donkey-anti-Mouse-IgG-H-L-Highly-Cross-Adsorbed-Secondary-Antibody-Polyclonal/A-21203
anti-rat IgG (H+L), Alexa Fluor 488 A-21208 Invitrogen 1:200 https://www.thermofisher.cn/cn/zh/antibody/product/Donkey-anti-Rat-IgG-H-L-Highly-Cross-Adsorbed-Secondary-Antibody-Polyclonal/A-21208?adobe_mc=MCMID%7C24401428113724416100518524426231235389%7CMCAID%3D3423F6676A295728-40000FA361BF316C%7CMCORGID%3D5B135A0C5370E6B40A490D44%40AdobeOrg%7CTS=1614293705
anti-rabbit IgG (H+L), Alexa Fluor Plus 647 A-31573 Invitrogen 1:200 https://www.thermofisher.cn/cn/zh/antibody/product/Donkey-anti-Rabbit-IgG-H-L-Highly-Cross-Adsorbed-Secondary-Antibody-Polyclonal/A-31573
Hoechst C1017 Beyotime 1:200 https://www.beyotime.com/product/C1017.htm
DAPI 4083 Cell Signaling Technology 1:200 https://www.cellsignal.cn/products/buffers-dyes/dapi/4083

Validation

All primary antibodies used in this study were purchased from commercial suppliers and have been validated by the manufacturers.

Eukaryotic cell lines

Policy information about cell lines and Sex and Gender in Research

Cell line source(s)

Primary Human Retinal Microvascular Endothelial Cells (ACBRI 181) were from Cell Systems; HEK293T cells (CRL-3216) AND HT1080 cells (CCL-121) were from ATCC.

Authentication

No further in-house authentication was performed for these cells.

Mycoplasma contamination

The cells were tested negative for mycoplasma contamination.

Commonly misidentified lines
(See ICLAC register)

None.

Animals and other research organisms

Policy information about studies involving animals; ARRIVE guidelines recommended for reporting animal research, and Sex and Gender in Research

Laboratory animals

The Kif11-flox mice (Strain S-CKO-03251, Cyagen) and Ctnnb1-flox mice (VSM4101958, Viewsolid) were crossed with Pdgfb-iCre-ER mice62 to obtain vascular endothelial cell-specific Kif11 or Ctnnb1 knockout mice, namely Kif11loxp/loxp; Pdgfb-iCre-ER (Kif11 cKO) and Ctnnb1loxp/loxp; Pdgfb-iCre-ER (Ctnnb1 cKO). Mice were induced through daily intraperitoneal injection with 50 μg tamoxifen (T6906, Topscience) from postnatal day 1 for 3 consecutive days. Tspan12 knockout (Tspan12 KO, Strain S-KO-08729, Cyagen) mice were used for single-cell RNA sequencing analysis. All mice were on a C57BL/6 background. The mice were maintained in an SPF animal facility under a controlled environment with a 12-hour light/dark cycle, free access to food and water, at Sichuan Provincial People's Hospital. The ambient temperature was maintained at 22–26°C with a relative humidity of 40–70%.

Wild animals

This study did not involve wild animals

Reporting on sex

Experiments were performed using mice of both sexes.

Field-collected samples

Not applicable.

Ethics oversight

All animal experiment procedures were approved by the Animal Protection and Use Committee of Sichuan Provincial People's Hospital (No.465, 2021) and were conducted in accordance with ethical standards, following the guidelines set by the Chinese Animal Welfare Committee for the care and use of laboratory animals.

Note that full information on the approval of the study protocol must also be provided in the manuscript.

Plants

Seed stocks	*Report on the source of all seed stocks or other plant material used. If applicable, state the seed stock centre and catalogue number. If plant specimens were collected from the field, describe the collection location, date and sampling procedures.*
Novel plant genotypes	*Describe the methods by which all novel plant genotypes were produced. This includes those generated by transgenic approaches, gene editing, chemical/radiation-based mutagenesis and hybridization. For transgenic lines, describe the transformation method, the number of independent lines analyzed and the generation upon which experiments were performed. For gene-edited lines, describe the editor used, the endogenous sequence targeted for editing, the targeting guide RNA sequence (if applicable) and how the editor was applied.*
Authentication	*Describe any authentication procedures for each seed stock used or novel genotype generated. Describe any experiments used to assess the effect of a mutation and, where applicable, how potential secondary effects (e.g. second site T-DNA insertions, mosiacism, off-target gene editing) were examined.*
